# Coordination of stress signals by the lysine methyltransferase SMYD2 promotes pancreatic cancer

**DOI:** 10.1101/gad.275529.115

**Published:** 2016-04-01

**Authors:** Nicolas Reynoird, Pawel K. Mazur, Timo Stellfeld, Natasha M. Flores, Shane M. Lofgren, Scott M. Carlson, Elisabeth Brambilla, Pierre Hainaut, Ewa B. Kaznowska, Cheryl H. Arrowsmith, Purvesh Khatri, Carlo Stresemann, Or Gozani, Julien Sage

**Affiliations:** 1 Department of Biology, Stanford University, Stanford, California 94305, USA;; 2 Institut Albert Bonniot, U1209, Institut National de la Santé et de la Recherche Médicale, UMR5309, Centre National de la Recherche Scientifique, Université Grenoble-Alpes, F-38700 Grenoble, France;; 3 Department of Pediatrics, Stanford University School of Medicine, Stanford, California 94305, USA;; 4 Department of Genetics, Stanford University School of Medicine, Stanford, California 94305, USA;; 5 Global Drug Discovery, Bayer Pharma AG, 13353 Berlin, Germany;; 6 Department of Medicine, Stanford University School of Medicine, Stanford, California 94305, USA;; 7 Institute for Immunity, Transplantation, and Infection, Stanford University School of Medicine, Stanford, California 94305, USA;; 8 Faculty of Medicine, Centre for Innovative Research in Medical and Natural Sciences, University of Rzeszów, 35959 Rzeszów, Poland;; 9 Structural Genomics Consortium, Princess Margaret Cancer Centre, University of Toronto, Toronto, Ontario M5G 2M9, Canada;; 10 Department of Medical Biophysics, University of Toronto, Toronto, Ontario M5G 2M9, Canada

**Keywords:** Ras, SMYD2, MAPKAPK3, pancreatic cancer, lung adenocarcinoma, lysine methylation

## Abstract

Pancreatic ductal adenocarcinoma (PDAC) is a lethal form of cancer with few therapeutic options. We found that levels of the lysine methyltransferase SMYD2 (SET and MYND domain 2) are elevated in PDAC and that genetic and pharmacological inhibition of SMYD2 restricts PDAC growth. We further identified the stress response kinase MAPKAPK3 (MK3) as a new physiologic substrate of SMYD2 in PDAC cells. Inhibition of MAPKAPK3 impedes PDAC growth, identifying a potential new kinase target in PDAC. Finally, we show that inhibition of SMYD2 cooperates with standard chemotherapy to treat PDAC cells and tumors. These findings uncover a pivotal role for SMYD2 in promoting pancreatic cancer.

## Abstract (precis)

Here, Reynoird et al. identify the protein lysine methyltransferase SMYD2 as a key regulator of pancreatic cancer. They demonstrate that SMYD2 levels are increased in PDAC, genetic and pharmacological inhibition of SMYD2 restricts PDAC growth, and the stress response kinase MAPKAPK3 (MK3) is a substrate of SMYD2 in PDAC cells.

Protein lysine methylation has emerged as a key cell signaling mechanism important for regulating physiologic and pathologic processes, including cancer (Black et al. 2012; Hamamoto et al. 2015). SMYD2 (SET and MYND domain 2) is a lysine methyltransferase (KMT) that has been implicated in cancer development. SMYD2 protein levels are elevated in various cancer types, and overexpression of SMYD2 in cancer cell lines promotes phenotypes associated with oncogenic transformation (Komatsu et al. 2009, 2015; Hamamoto et al. 2014; Sakamoto et al. 2014; Liu et al. 2015). As a KMT, SMYD2 is a monomethyltransferase that was first thought to catalyze methylation of histone H3 at Lys36 (H3K36me) (Brown et al. 2006), but it is unlikely that SMYD2 has this activity in cells. Six nonhistone substrates of SMYD2 have been validated thus far in cells: the tumor suppressor p53 at Ly370 (p53K370me1), the tumor suppressor RB at two sites (RBK810me1 and RBK860me1) (Huang et al. 2006; Saddic et al. 2010; Cho et al. 2012), HSP90, ERα, PARP1, and PTEN (Abu-Farha et al. 2008; Zhang et al. 2013; Piao et al. 2014; Nakakido et al. 2015). A recent proteomics study identified a number of additional candidate SMYD2 targets, including the AHNAK and AHNAK2 proteins, which have been involved in cell migration and invasion (Olsen et al. 2016). Thus, all of the known SMYD2-generated methylation events may play a role in cancer. Despite these intriguing links, however, a direct in vivo role for SMYD2 in tumorigenesis has not been tested.

Here we investigate the function of SMYD2 in the lethal cancer pancreatic ductal adenocarcinoma (PDAC) using mouse and cellular models. We found that SMYD2 normally promotes Ras-driven development of PDAC. Notably, loss of SMYD2 correlates with diminished inflammation in PDAC, and we identified the stress response kinase MAPKAPK3 as a new and physiologically relevant SMYD2 substrate. Finally, inhibition and depletion of SMYD2 enhances the efficacy of chemotherapeutics. Together, our findings suggest new roles for SMYD2 in inflammation and stress responses and identify SMYD2 and MAPKAPK3 as potential therapeutic targets to treat pancreatic cancer.

## Results

### SMYD2 is required for efficient PDAC development in mice

SMYD2 plays an important role in embryonic stem cell biology and skeletal and cardiac muscle function but is dispensable for heart development (Diehl et al. 2010; Donlin et al. 2012; Sese et al. 2013; Sajjad et al. 2014). Beyond these activities, normal physiological roles for SMYD2 in vivo remain largely unknown. To investigate the role of SMYD2 in the pancreas and PDAC, we used *Smyd2*^*loxP/loxP*^ conditional mutant mice. Deletion of *Smyd2* specifically in the pancreata of mice (*Ptf1a*^+/*Cre*^;*Smyd2*^*loxP/loxP*^) (Supplemental Fig. S1A) resulted in no apparent developmental consequences and no physiological defects, as determined by the expression of key pancreatic markers for functional acinar cells (amylase) and endocrine cells (insulin and glucagon) (Supplemental Fig. S1B–D). Mice with whole-body deletion of the *Smyd2* gene were also fully viable and fertile (data not shown). Thus, SMYD2 is dispensable for mice under the conditions that we tested.

**Figure 1.**
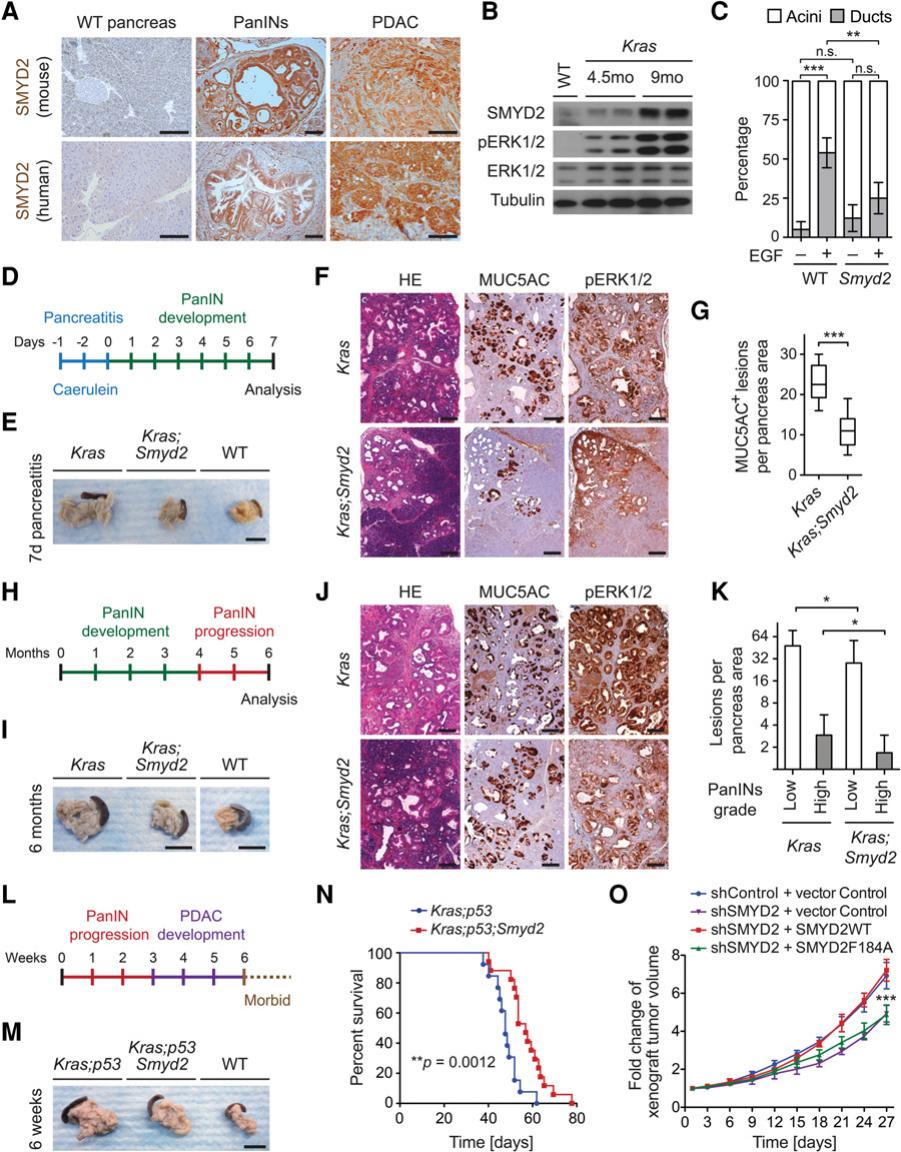
SMYD2 promotes Ras-driven pancreatic cancer. (*A*) Representative immunohistochemical analysis of SMYD2 expression (12 independent samples examined) from mouse and human wild-type (WT) pancreata, PanIN lesions, and PDAC tumors. The mouse tumors were obtained from *Kras* mutant mice. Bars, 100 μm. (*B*) Immunoblot analysis with the indicated antibodies on pancreas lysates from wild-type mice and *Kras* mutant mice at 4.5 and 9 mo of age (two biological replicates). Tumor progression is accompanied by increased ERK1/2 phosphorylation (pERK1/2). Tubulin and ERK1/2 served as loading controls. (*C*) Quantification of wild-type and *Smyd2* mutant mouse acinar clusters undergoing acinar-to-ductal metaplasia (ADM) and forming ducts ex vivo in response to coculture with EGF or vehicle control for 3 d (four independent biological replicas with three technical replicas each). (*D*) Schematic of the pancreatitis-induced precancerous lesion formation protocol in *Kras* mutant mice. (*E*) Representative examples of pancreata from wild-type (*n* = 3), *Kras* mutant (*n* = 5), and *Kras;Smyd2* mutant (*n* = 5) mice 7 d after pancreatitis induction. Bars, 1 cm. (*F*) Representative images of hematoxylin and eosin (HE), MUC5AC (a marker of PanIN lesions), and pERK1/2 immunohistochemistry (a marker of Ras pathway activity) from *Kras* and *Kras;Smyd2* mutant mice 7 d after pancreatitis induction. Bars, 100 μm. (*G*) Quantification of MUC5AC^+^ lesions in caerulein-treated pancreata from *Kras* and *Kras;Smyd2* mutant mice. (*H*) Schematic of the spontaneous pancreas tumor progression model in *Kras* mutant mice. (*I*) Representative HE staining and immunohistochemistry for MUC5AC and pERK1/2 in *Kras* (*n* = 12) and *Kras;Smyd2* (*n* = 9) mutant mice (6 mo of age). Bars, 100 μm. (*J*) Representative examples of the pancreata of *Kras* (*n* = 12) and *Kras;Smyd2* (*n* = 9) mutant mice (6 mo of age). Bars, 1 cm. (*K*) Quantification of spontaneous PanIN lesions formed in *Kras* and *Kras;Smyd2* mutant mice. The grades of the lesions are indicated. (*L*) Schematic of the spontaneous pancreas tumor progression model in *Kras;p53* mutant mice. (*M*) Representative examples of the pancreata of *Kras;p53* (*n* = 5) and *Kras;p53;Smyd2* (*n* = 5) mutant mice (6 wk of age). Bars, 1 cm. (*N*) Kaplan-Meier survival curves of *Kras;p53* (*n* = 13, median survival = 57 d) and *Kras;p53;Smyd2* (*n* = 17, median survival = 68 d) mutant mice. (**) *P* = 0.0012 by log-rank test for significance. (*O*) Xenograft tumor volume analysis for SW1990 human PDAC cells growing subcutaneously in immunocompromised mice with a control knockdown or SMYD2 knockdown, rescued with control, wild-type SMYD2, or catalytically dead SMYD2-F184A. *n* = 2 for shSMYD2 + vector control; *n* = 6 for the other conditions. The *P*-value is for rescue with wild-type SMYD2 versus SMYD2-F184A. (n.s.) Not significant; (*) *P* < 0.05; (**) *P* < 0.01; (***) *P* < 0.001, *P*-value calculated by two-tailed unpaired Student's *t*-test. Data are represented as mean ± standard error of the mean (SEM).

A meta-analysis of eight publicly available human PDAC data sets showed consistent up-regulation of *SMYD2* mRNA levels (Supplemental Fig. S1E). Analysis of The Cancer Genome Atlas (TCGA) data sets further revealed that the *SMYD2* locus is frequently amplified in human PDAC tumors (Supplemental Fig. S1F). Moreover, while SMYD2 protein expression was undetectable by immunohistochemistry in the normal pancreas, it was clearly observed in sections from murine and human pancreatic intraepithelial neoplasia (PanIN) and PDAC samples (Fig. 1A). Expression of K-Ras^G12D^ from the endogenous *Kras* locus in *Ptf1a*^+/*Cre*^;*Kras*^+/*LSL-G12D*^ mice is well known to trigger tumor formation in the pancreas (Hingorani et al. 2003). Immunoblots from the pancreata of these *Kras* mutant mice showed that SMYD2 levels increased with cancer progression (Fig. 1B). These results led us to investigate possible functional roles of SMYD2 in pancreatic tumorigenesis.

Acinar-to-ductal metaplasia (ADM) is an early step in PDAC initiation triggered upon activation of Ras signaling (Guerra et al. 2003; Zhu et al. 2007). We found that *Smyd2* deletion significantly decreased ADM from isolated acinar cells ex vivo in response to EGF stimulation (Fig. 1C). PanIN lesions can be induced by the repeated injections of caerulein into young *Kras* mutant mice (Morris et al. 2010). In this system, *Smyd2* deletion reduced the development of PanINs, as determined by pancreas volume and quantitative analysis of the PanIN marker MUC5AC (Fig. 1D–G). Loss of SMYD2 led to decreased proliferation (Ki67) but did not significantly affect apoptotic cell death (cleaved Caspase 3) in this assay (Supplemental Fig. S2A–C). In the absence of caerulein injection, *Kras* mutant mice develop low- and high-grade PanIN lesions by 6 mo (Hingorani et al. 2003). In this context, *Smyd2* loss again attenuated the development of precancerous lesions, as determined by the analysis of key markers of Ras pathway activation (pERK1/2 [ERK1/2 phosphorylation]), proliferation (Ki67), apoptosis (cleaved Caspase 3), and stromal response (αSMA) (Fig. 1H–K; Supplemental Fig. S2D–H). Survival studies of Ras-driven PDAC are commonly conducted in a p53 mutant background (*Ptf1a*^+/*Cre*^;*Kras*^+/*LSL-G12D*^; *Trp53*^*loxP/loxP*^), where aggressive tumors result in rapid death from cancer (Bardeesy et al. 2006). SMYD2 loss in this context impeded gross cancer development and extended the life span of *Kras;p53* mutant mice by 17% (Fig. 1L–N; Supplemental Fig. S2I–K). Notably, these protumorigenic functions of SMYD2 likely require its catalytic activity, as the growth of human PDAC xenografts depleted of endogenous SMYD2 was restored to normal growth rates by complementation with wild-type SMYD2 but not a catalytic-dead SMYD2 mutant, which behaved like the SMYD2 depletion (Fig. 1O; Supplemental Fig. S3A).

Overall, these results indicate that SMYD2 is required for the efficient transformation of pancreatic cells by oncogenic K-Ras in vivo, supporting the idea that targeting the enzymatic activity of SMYD2 may be therapeutic in PDAC. We note that p53 plays a key role in PDAC suppression (Bardeesy et al. 2006), and SMYD2 methylates and inactivates p53 (Huang et al. 2006), suggesting that this signaling module is likely important for SMYD2 functions in PDAC. However, our studies using *p53*-deleted mice (Fig. 1L–N) clearly indicate that SMYD2 also has an important p53-independent role in PDAC. We performed similar experiments in a mouse model of lung adenocarcinoma driven by oncogenic K-Ras and loss of p53 and again found a key role for SMYD2 in the development of these tumors (Supplemental Fig. S4). Thus, inhibiting SMYD2 likely impacts on multiple pathways that are important for Ras-driven pancreatic cancer and lung adenocarcinoma.

### SMYD2 monomethylates MAPKAPK3 at Lys355

**Figure 2.**
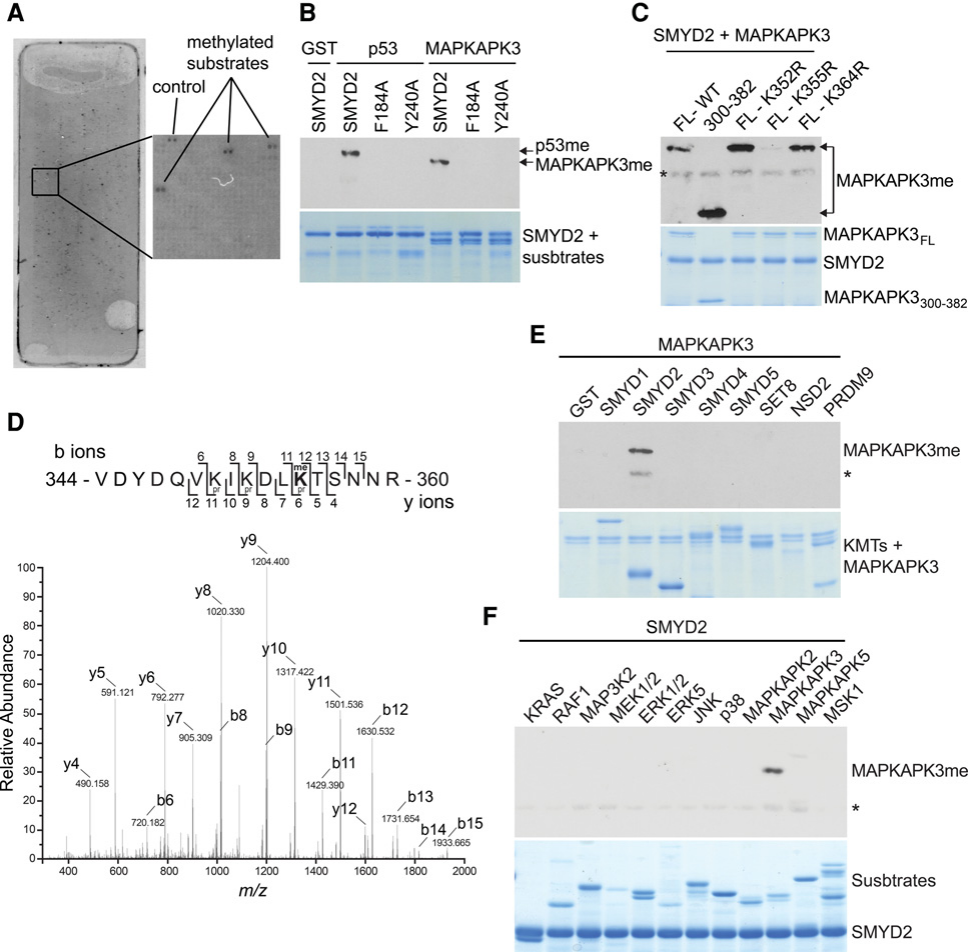
SMYD2 monomethylates MAPKAPK3 at Lys355. (*A*) SMYD2 methylation on a protein array platform identified candidate substrates. A representative image (*n* = 2 independent experiments) shows a SMYD2 methylation assay on a ProtoArray. The *right* panel is a close-up of the indicated space on the array. (*B*) SMYD2 directly methylates MAPKAPK3. An in vitro methylation assay on full-length recombinant MAPKAP3, p53 (as a positive control), and GST (as a negative control) with recombinant wild-type (WT) SMYD3 or the indicated catalytic mutants in a representative radiolabeled methylation assay is shown. *n* = 5. (*Top* panel) Autoradiogram of a methylation assay. (*Bottom* panel) Coomassie stain of proteins in the reaction. (*C*) SMYD2 methylates MAPKAPK3 at K355. In vitro methylation assays as in *B* with the indicated MAPKAPK3 mutants or truncation are shown (full-length [FL], truncated [300–382], and point mutants K352R, K355R, and K364R). *n* = 2. An asterisk indicates SMYD2 automethylation. (*D*) SMYD2 monomethylates MAPKAPK3 at K355. Tandem mass spectrometry (MS/MS) spectrum identifying monomethylated K355 present on the MAPKAPK3 peptide after in vitro SMYD2 methylation of MAPKAPK3. Note that deuterated S-adenosyl-l-methionine was used as a methyl donor and that samples were chemically propionylated prior to trypsin digestion (see the Materials and Methods). *n* = 2. (*E*) MAPKAPK3 is a specific substrate of SMYD2. In vitro methylation assays as in *B* on MAPKAPK3 using the indicated KMTs are shown. *n* = 2. GST was used as a negative control. An asterisk indicates SMYD2 automethylation. (*F*) SMYD2 specifically methylates MAPKAPK3. In vitro SMYD2 methylation assays as in *B* on the indicated 12 different MAPK pathway-related proteins are shown. *n* = 3. An asterisk indicates SMYD2 automethylation.

We used a protein array platform to identify potential SMYD2 substrates that, in addition to p53, may be involved in PDAC expansion (Levy et al. 2011). Based on two independent replicas, 159 of the ∼9500 proteins present on the array were identified as SMYD2 substrates in this in vitro system (Fig. 2A; Supplemental Table S1). Relative to other KMTs, SMYD2 is recognized to have robust in vitro activity and methylate several substrates, including H3K36 (Zhang et al. 2015); however, in our analysis, we did not observe physiologically relevant methylation at H3K36 by SMYD2 (N Reynoird and O Gozani, unpubl.). We identified only HSP90 among the previously known targets in our list of 159 candidate hits. PTEN was not methylated in this system, whereas p53, RB, ERα, and PARP1 are not present on the protoarrays. We chose to follow up potential new substrates that met the following criteria to point toward candidate therapeutic targets: (1) implicated in cancer, (2) linked to Ras signaling (a key driver of PDAC), and (3) amenable to small molecule inhibition. These criteria restricted the list to four kinases, PAK4, PLK1, AURKA, and MAPKAPK3. Out of these four candidates, only MAPKAPK3 (also named MK3 or 3PK) was confirmed as a substrate of SMYD2 in in vitro methylation assays (Fig. 2B; data not shown).

MAPKAPK3 is a MAPK-activated protein kinase that can be directly phosphorylated by ERK1/2, p38 kinase, and SAPK/JNK (Ludwig et al. 1996; McLaughlin et al. 1996). MAPKAPK3 is involved in inflammation and stress responses (Gaestel 2006, 2013; Ronkina et al. 2007, 2010; Kyriakis and Avruch 2012; Moens et al. 2013). Notably, in our PDAC models, depletion of *Smyd2* resulted in a decreased stromal response, which is associated with diminished inflammation (Supplemental Fig. S2D,H). Moreover, by immunoblot analysis, SMYD2 and MAPKAPK3 were detected primarily in the cytoplasm of human and murine PDAC cell lines (Supplemental Fig. S3B,C). Together, these results suggest that MAPKAPK3 could be a cancer-relevant cytoplasmic target of SMYD2.

Recombinant wild-type SMYD2, but not two catalytically dead mutants, methylated recombinant MAPKAPK3 and p53 as a positive control in vitro (Fig. 2B). Using a mutagenesis approach, we identified Lys355 of MAPKAPK3 as the site of methylation catalyzed by SMYD2 (Fig. 2C). Liquid chromatography-tandem mass spectrometry (LC-MS/MS) analysis of MAPKAPK3 methylated by SMYD2 in vitro identified monomethylated K355 but no dimethylation or trimethylation (Fig. 2D; Supplemental Fig. S5A). We used deuterated S-adenosyl-methionine (SAM; which increases the mass of me1 from 14.016 Da to 17.034 Da) in the MS experiments to rule out possible artifactual chemical methylation (see the Materials and Methods) (Jung et al. 2008). SMYD2 was the only KMT of the eight active enzymes that we tested that could methylate MAPKAPK3 in vitro (Fig. 2E). Furthermore, SMYD2, besides MAPKAPK3, had little activity on the related kinase MAPKAPK5 and no detectable activity on 10 other members of MAPK signaling cascades (Fig. 2F). Note that the related protein MAPKAPK2, which shares ∼75% sequence identity with MAPKAPK3 (Ronkina et al. 2008), does not have a lysine at the position equivalent to K355 and is not methylated by SMYD2 (Fig. 2F; Supplemental Fig. S5B). Thus, SMYD2 catalyzes monomethylation of MAPKAPK3 at K355 (MAPKAPK3-K355me1) in vitro.

**Figure 3.**
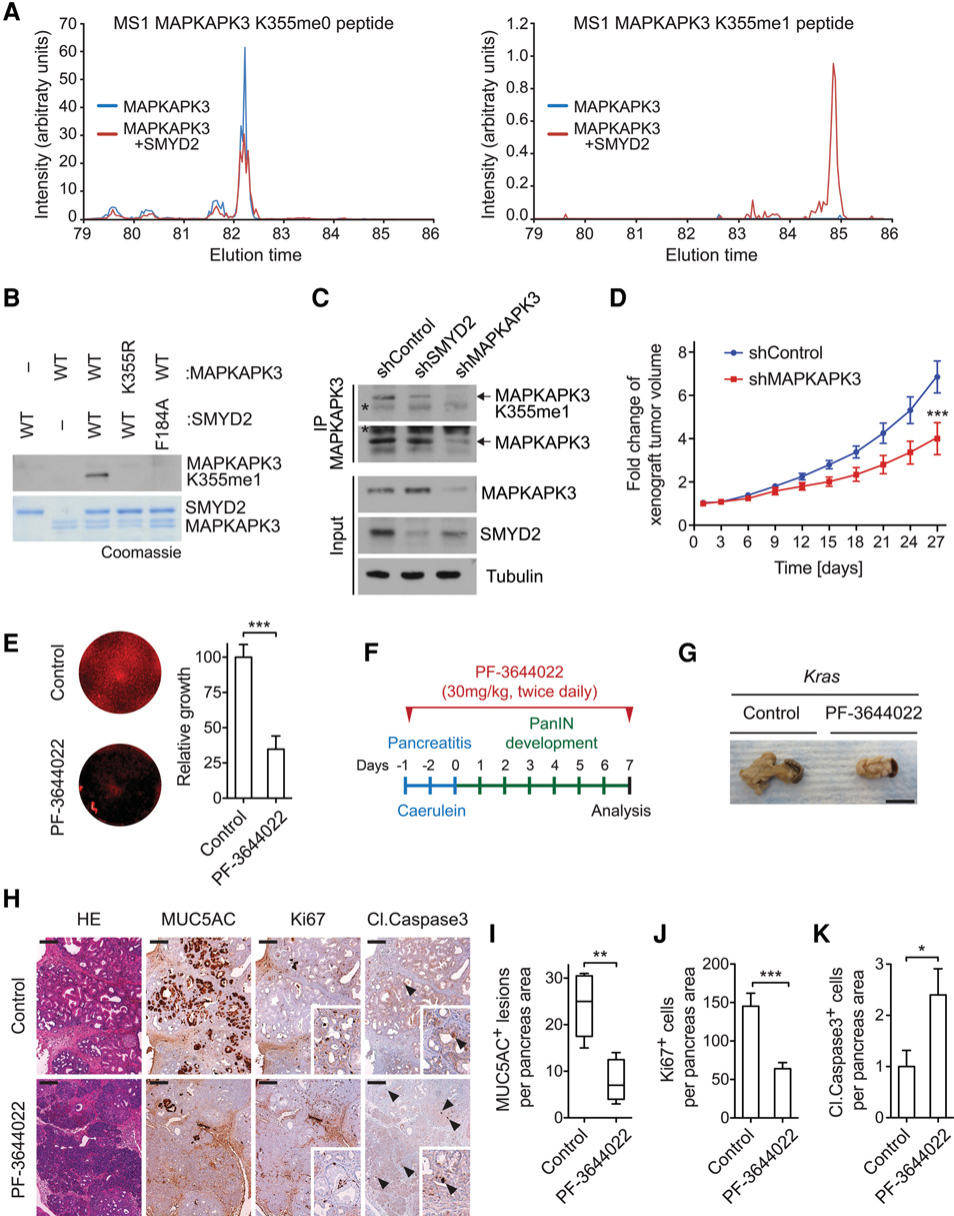
MAPKAPK3 is methylated in PDAC cells and regulates PDAC-associated phenotypes. (*A*) SMYD2 induces MAPKAPK3-K355me1 in cells. High-performance liquid chromatography (HPLC) elution profiles show a K355-containing peptide that is unmethylated (*left* panel) and methylated (*right* panel) from HEK293T cells overexpressing either MAPKAPK3 alone (blue line) or MAPKAPK3 and SMYD2 (red line) (*n* = 2). See Supplemental Figure S4C for the MS/MS spectrum of MAPKAPK3-K355me1. (*B*) Characterization of the MAPKAPK3 K355me1 antibody. Immunoblot analysis with the anti-MAPKAPK3-K355me1 antibody on the product of in vitro methylation assays using combinations of either wild-type or mutant forms of recombinant SMYD2 and MAPKAPK3, as indicated. The Coomassie gel is shown as loading control. *n* = 3. (*C*) SMYD2 is required for methylation of MAPKAP3 in cells. An immunoblot analysis with the indicated antibodies after MAPKAPK3 immunoprecipitation using extracts from SW1990 cells stably expressing control, SMYD2, or MAPKPAK3 shRNAs is shown. Inputs are shown as loading and shRNA efficiency controls. *n* = 2. An asterisk indicates nonspecific bands. (*D*) MAPKAPK3 promotes pancreatic cancer cell growth in a xenograft model. Xenografts tumor volume analysis for SW1990 human PDAC cells growing subcutaneously in immunocompromised mice with a control knockdown or MAPKAPK3 knockdown. *n* = 6 tumors for each experimental group. (*E*) Inhibition of MAPKAPK2/3/5 attenuates the growth of human PDAC cells. SW1990 cells were treated with DMSO (control) or 1 μM MAPKAPK2/3/5 inhibitor PF-3644022 for 9 d. After drug treatment, cells were stained with Sapphire 700 (*left* panel), and the fluorescence signal was quantified (*right* panel). (*F*) Schematic of the pancreatitis-induced precancerous lesion formation protocol in *Kras* mutant mice and treatment protocol with the PF-3644022 inhibitor. (*G*) Representative pancreata images in control mice and mice treated with the PF-3644022 inhibitor 7 d after pancreatitis induction. *n* = 5 for each experimental group. Bars, 1 cm. (*H*) Representative images of HE staining and immunohistochemistry for MUC5AC, Ki67, and cleaved Caspase 3 in *Kras* mutant mice treated with vehicle (control) or the PF-3644022 inhibitor 7 d after pancreatitis induction. Bars, 100 μm. (*I–K*) Quantification of MUC5AC-, Ki67-, and cleaved Caspase 3-positive cells in caerulein-treated pancreata from *Kras* mutant mice treated with vehicle control (*n* = 5) or the PF-3644022 inhibitor (*n* = 5) 7 d after pancreatitis induction. (*) *P* < 0.05; (**) *P* < 0.01; (***) *P* < 0.001, *P*-value calculated by two-tailed unpaired Student's *t*-test. Data are represented as mean ± SEM.

To investigate methylation of MAPKAPK3 in cells, we coexpressed SMYD2 and MAPKAPK3 in HEK293T cells. Flag immunoprecipitation followed by in-gel tryptic digest and LC-MS/MS identified MAPKAPK3-K355me1 in a SMYD2-dependent manner, indicating that SMYD2 can methylate MAPKAPK3 in cells (Fig. 3A; Supplemental Fig. S5C). Next, after screening multiple candidate anti-methyl antibodies, we identified an antibody that specifically recognized MAPKAPK3 methylated at K355me1 (Fig. 3B; see the Materials and Methods). Endogenous methylation of MAPKAPK3 at K355 was observed in human SW1990 PDAC cells as well as human H358 and H441 lung adenocarcinoma cells, and this signal decreased upon RNAi-mediated depletion of SMYD2 (Fig. 3C; Supplemental Fig. S5D). Together, these data identify MAPKAPK3 as an in vitro and in vivo substrate of SMYD2 and indicate that SMYD2 activity maintains physiological levels of MAPKAPK3-K355me1 in PDAC cells.

### MAPKAPK3 inhibition and PDAC development

Hyperinflammation has been linked to pancreatic cancer development (Guerra et al. 2007; Gukovsky et al. 2013). MAPKAPK3 and its family member, MAPKAPK2, are both implicated in stress responses and inflammation (Wysk et al. 1999; Sassano et al. 2005; Ronkina et al. 2011; Wei et al. 2015). Moreover, SMYD2 was recently shown to regulate IL-6 and TNFα production (Xu et al. 2015), and the concentration of a number of inflammatory cytokines is lower in the serum of *Kras;Smyd2* mutant mice relative to *Kras* mutant mice alone (Supplemental Fig. 6A). In addition, we recently found that MAP3K2, a kinase methylated by the SMYD2 family member SMYD3, regulates the expansion of PDAC cells (Mazur et al. 2014). Based on these observations, we postulated a role for MAPKAPK3 in the SMYD2-mediated promotion of PDAC development.

Consistent with this hypothesis, MAPKAPK3 depletion attenuated the growth of SW1990 xenografts (Fig. 3D; Supplemental Fig. S6B). A small molecule approach was used to test the role of MAPKAPK3 by an independent method. While there is no specific MAPKAPK3 inhibitor, the compound PF-3644022 targets MAPKAPK3 and the related proteins MAPKAPK2 and MAPKAPK5 (Mourey et al. 2010; Moens et al. 2013). Treatment with PF-3644022 suppressed the expansion of SW1990 cells in culture (Fig. 3E). In addition, the development of PDAC in *Kras* mutant mice treated with caerulein to induce pancreatitis was attenuated by PF-3644022 treatment (Fig. 3F,G). The treatment led to fewer PanIN lesions, decreased cellular proliferation, increased apoptotic cell death, a decreased number of immune cells, a diminished stromal response, lower activation of the Ras pathway, and decreased production of inflammatory cytokines (Fig. 3H–K; Supplemental Fig. S6C–F). These data suggest a model in which SMYD2 activates MAPKAPK3, which may contribute to the promotion of Ras-driven PDAC development by SMYD2 and support a role for the MAPKAPK3 kinase and its family members in PDAC.

Methylation events generally function through the modulation of protein–protein interactions (Wilkinson and Gozani 2014). We sought to identify candidate methyl-sensitive binding partners of MAPKAPK3-K355me1. We took both candidate approaches and unbiased approaches such as using stable isotope labeling by amino acids in cell culture (SILAC)-based quantitative proteomic screening to isolate proteins that bound differentially to unmodified MAPKAPK3-K355 peptides versus MAPKAPK3-K355me1 peptides. However, we were not able to identify a specifically regulated interaction (data not shown), and future work will be needed to elucidate the molecular consequences of K355 methylation in regulating MAPKAPK3 biological and pathologic functions.

### SMYD2 inhibition enhances the effects of chemotherapy in PDAC

**Figure 4.**
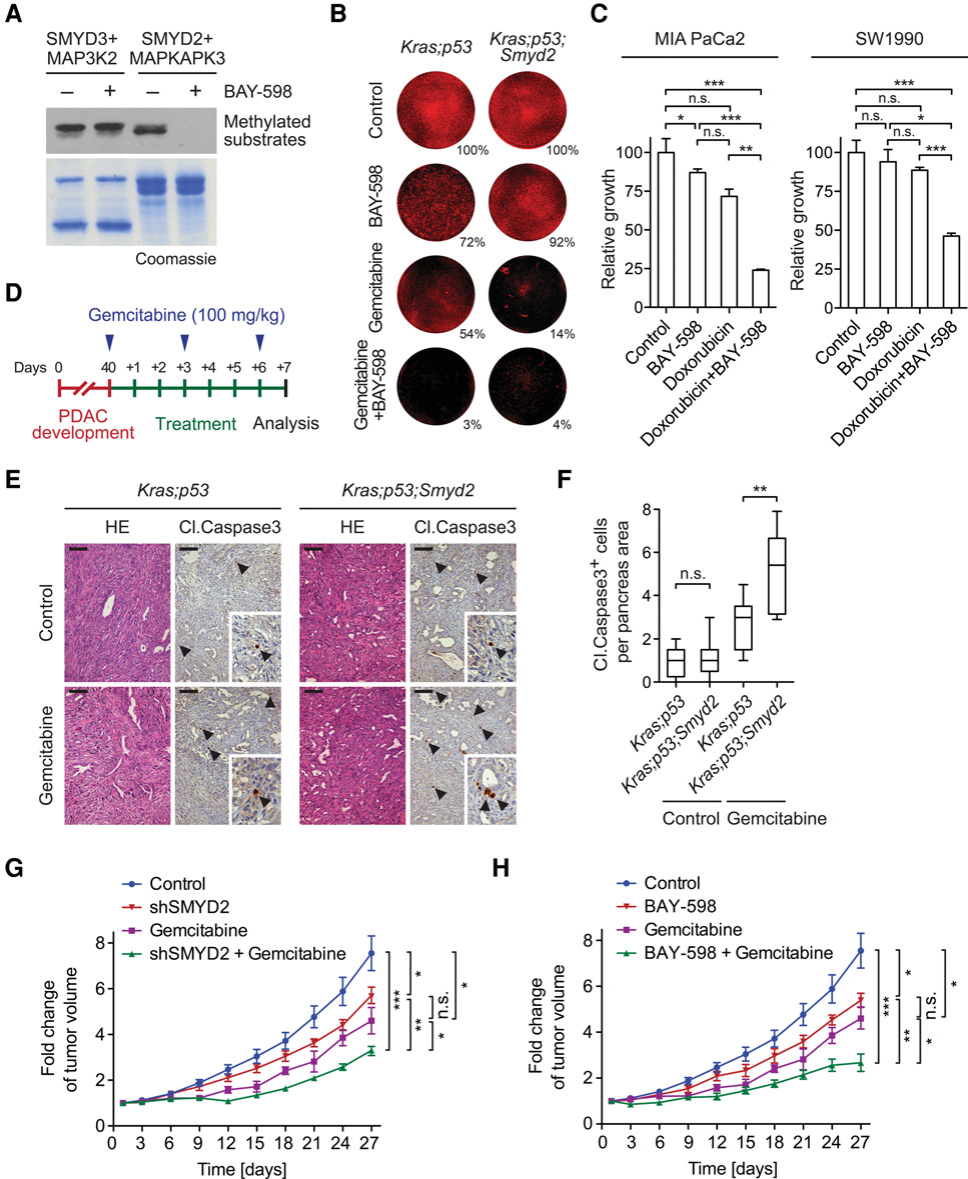
SMYD2 inhibition enhances PDAC chemosensitivity in vitro and in vivo. (*A*) BAY-598 inhibits SMYD2. An immunoblot analysis of methylation assays (of MAP3K2 by SMYD3 and MAPKAPK3 by SMYD2) with the SMYD2 inhibitor BAY-598 is shown. *n* = 3. A Coomassie stain of the proteins in the reaction is shown in the *bottom* panel. (*B*) Cotreatment with BAY-598 and gemcitabine inhibits the expansion of PDAC cells from low density. Representative images of the response of primary cancer cell lines established form *Kras;p53* and *Kras;p53;Smyd2* mutant mice to treatment with DMSO (control), 10 μM BAY-598, and 0.1 μM gemcitabine for 9 d are shown. *n* = 2 independent experiments performed in triplicates. One example of Sapphire 700 staining is shown, and the average value of the triplicate for that experiment is shown. (*C*) BAY-598 enhances the toxicity of doxorubicin. Changes in cell viability (MTT assay) in response to treatment with 10 μM BAY-598, 1 μM doxorubicin, and cotreatment for 48 h are shown. Combined results of two independent experiments performed in triplicates are shown. (*D*) Schematic protocol for acute gemcitabine treatment protocol in *Kras;p53* mutant mice with advanced PDAC. (*E*) Representative images for HE and cleaved Caspase 3 immunohistochemistry (arrowheads) in controls and gemcitabine-treated PDAC tumors. *n* = 5 for each experimental group. Bars, 100 μm. (*F*) Quantification of cleaved Caspase 3-positive cells in PDAC tumors from the indicated genotypes of mice treated with gemcitabine. *n* = 5 for each experimental group. (*G*,*H*) Tumor volume quantification of human SW1990 PDAC cells growing subcutaneously in immunocompromised mice following SMYD2 inactivation and chemotherapy. (*G*) SMYD2 knockdown (control cells and empty vector). (*H*) SMYD2 inhibition by 50 mg/kg BAY-598 once daily. Mice were treated with 100 mg/kg gemcitabine every third day (*G*,*H*) or vehicle control (*G*,*H*). *n* = 5 mice for each treatment group in one experiment. (*) *P* < 0.05; (**) *P* < 0.01; (***): *P* < 0.001, *P*-value calculated by two-tailed unpaired Student's *t*-test. Data are represented as mean ± SEM.

Chemotherapy remains the “standard of care” approach in PDAC patients even though it is, unfortunately, only marginally effective. One mechanism for cancer cells to combat chemotherapy is to use a protective stress response (Jaramillo and Zhang 2013; Sui et al. 2013; Palam et al. 2015). The validated SMYD2 targets (i.e., RB, p53, HSP90, PARP1, PTEN, HSP90, and MAPKAPK3) are involved at some level in stress signaling, which led us to postulate that activation of SMYD2 might represent one mechanism regulating the response of PDAC cells to chemotherapy. To test this idea, we used the highly selective SMYD2 small molecule inhibitor BAY-598. As expected, BAY-598 treatment blocked in vitro methylation of MAPKAPK3 by SMYD2 but had no activity against the SMYD2-related KMT SMYD3, which methylated its substrate, MAP3K2, irrespective of the presence of BAY-598 (Fig. 4A; Mazur et al. 2014). BAY-598 treatment reduced the growth of *Kras;p53* mutant PDAC cells after 9 d in culture but had little impact on the growth of *Kras;p53;Smyd2* mutant cells (Fig. 4B), further supporting the specificity of this small molecule inhibitor of SMYD2 as well as a role for SMYD2 in promoting the growth of PDAC cells. Notably, the chemotherapeutic gemcitabine was far more potent in inhibiting clonal expansion of *Kras;p53* mutant PDAC cells when administered in combination with BAY-598 or used to treat cells lacking SMYD2 (*Kras;p53;Smyd2*), suggesting that chemotherapy treatment and SMYD2 inhibition cooperate to target PDAC cells in this assay (Fig. 4B).

Human PDAC cell lines such as SW1990 and MIA PaCa2 cells are largely resistant to acute treatment (48 h or less) with chemotherapeutic agents like gemcitabine and doxorubicin and are only marginally inhibited by acute treatment with the SMYD2 inhibitor BAY-598. However, these PDAC cells were highly sensitive to acute treatment with a combination therapy consisting of low-dose treatment with doxorubicin and BAY-598 (Fig. 4C; Supplemental Fig. S6G). Based on these observations in cell culture, we next tested whether *Smyd2* deletion potentiated the effects of chemotherapy on advanced PDAC tumors in vivo. To this end, *Kras;p53* and *Kras;p53;Smyd2* mutant mice were aged for 40 d. At this time point, *Kras;p53* mutant mice survive only 15–20 d (Mazur et al. 2015). *Smyd2* deletion did not affect the number of apoptotic cells in *Kras;p53* mutant tumors (Fig. 4E,F; Supplemental Fig. S2), and gemcitabine treatment also had little to no effect on apoptosis in *Kras;p53* mutant mice, but the proapoptotic effects of this chemotherapeutic agent were enhanced in *Kras;p53;Smyd2* mutant tumors (Fig. 4E,F). Consistent with these observations, cotreatment of gemcitabine with either SMYD2 inhibition or its deletion attenuated the growth of SW1990 xenografts compared with single treatments (Fig. 4G,H). Thus, SMYD2 inhibition and gemcitabine treatment may potentially be combined in patients to improve the efficacy of chemotherapy.

## Discussion

In conclusion, SMYD2 is a KMT that is dispensable for the development of mice and the maintenance of homeostasis in adult mice, including in the pancreas, but whose expression is elevated in PDAC and other cancers. These observations led us to determine whether SMYD2 may be oncogenic in PDAC and thus constitute a new therapeutic target in this cancer type. Our data in mouse models and human xenografts uncover a pivotal role for SMYD2 in the promotion of PDAC development in vivo. SMYD2 shares a methyltransferase domain with SMYD3, a KMT that also promotes PDAC development (Mazur et al. 2014). Since they target different substrates, it is unlikely that these two distinct enzymes would compensate for each other, but the dual inactivation of SMYD2 and SMYD3 may inhibit PDAC (and lung adenocarcinoma) more potently than the inactivation of each enzyme alone.

To date, all of the validated methylated substrates of SMYD2 are implicated in stress responses and cellular checkpoints (i.e., RB, p53, HSP90, PARP1, PTEN, and HSP90) (Huang et al. 2006, 2007; Saddic et al. 2010; Abu-Farha et al. 2011; Cho et al. 2012; Zhang et al. 2013; Nakakido et al. 2015). Our studies identify a new substrate, MAPKAPK3, that is also implicated in the cellular response to stress and inflammation (Ronkina et al. 2008; Moens et al. 2013). Interestingly, both SMYD2 (Xu et al. 2015) and MAPKAPK3 (Wysk et al. 1999) have been linked to IL-6 and TNFα regulation. MAPKAPK3 and its closely related family member, MAPKAPK2 (which is not methylated by SMYD2), have also been implicated in DNA damage response and chemotherapy response (Morandell et al. 2013; Dietlein et al. 2015), and their common substrate, HSP27, has been shown to mediate the effects of gemcitabine toxicity in PDAC cells (Mori-Iwamoto et al. 2007; Kuramitsu et al. 2012). These observations and our data showing greater effects of chemotherapy upon inhibition of SMYD2 in PDAC cells raise the possibility that SMYD2 inhibitors and MAPKAPK2/3 inhibitors may be used in combination to enhance the effects of chemotherapy in PDAC patients. Thus, SMYD2 may promote cancer by orchestrating a cellular response to stress in cancer cells.

## Material and methods

### Ethics statement

All procedures involving animals were reviewed and approved by the Stanford University Administrative Panel on Laboratory Animal Care (Sage laboratory protocol no. 13565) and carried out in an Association for the Assessment and Accreditation of Laboratory Animal Care-accredited facility.

### Mouse strains

*Ptf1a*^+/*Cre*^, *Kras*^+/*LSL-G12D*^, *Trp53*^*loxP/loxP*^ mice have been described before (Jonkers et al. 2001; Kawaguchi et al. 2002; Hingorani et al. 2003). *Smyd2*^*tm1a(KOMP)Wtsi*^ mice were obtained from the Knockout Mouse Project (KOMP) repository. Details on the targeted allele are available on the KOMP Web site. Briefly, mice were constructed using the “knockout first” strategy. In this allele, insertion of a LacZ cassette with a strong splice acceptor in intron 2 of the *Smyd2* gene creates a knockout allele. Expression of the Cre recombinase in cells removes the LacZ cassette and further deletes several *Smyd2* exons, resulting in a null allele (Supplemental Fig. S1A). Mice were in a mixed C57BL/6;129/Sv background, and we systematically used littermates as controls in all of the experiments (sex ratio per cohort balanced). All animals were numbered, and experiments were conducted blinded. After data collection, genotypes were revealed, and animals were assigned to groups for analysis. For treatment experiments, mice were randomized. None of the mice with the appropriate genotype were excluded from this study. Histopathological analysis was conducted on de-identified slides.

### Pancreatic cancer mouse models

#### Pancreatitis-induced tumorigenesis

Acute pancreatitis was induced at 6 to 8 wk of age in *Ptf1a*^+/*Cre*^;*Kras*^+/*LSL-G12D*^ (*Kras*) mice by administration of eight hourly intraperitoneal injections of 100 µg of caerulein per kilogram of body weight (Sigma-Aldrich) over two consecutive days as described previously (Morris et al. 2010; Mazur et al. 2014). Mice were treated as indicated with 30 mg/kg MAPKAPK2/3 inhibitor PF-3644022 intraperitoneally twice daily or 10% vehicle (2-hydroxypropyl)-β-cyclodextrin (Sigma-Aldrich). Pancreatic lesions were analyzed 7 d after the last injection. In parallel, animals were analyzed at day 0 to confirm the caerulein-induced damage. None of the treatments affected the initial pancreatic injury (data not shown).

#### Spontaneous model of PanIN development

PanIN progression was analyzed in *Ptf1a*^+/*Cre*^;*Kras*^+/*LSL-G12D*^ (*Kras*) and *Ptf1a*^+/*Cre*^;*Kras*^+/*LSL-G12D*^; *Smyd2*^*loxP/loxP*^ (*Kras;Smyd2*) mice aged for 6 mo. Quantification of low-grade (PanIN1a and PanIN1b) and high-grade (PanIN2 and PanIN3) lesions was conducted on deidentified slides based on the classification consensus (Hruban et al. 2006). Five images (100×) were taken in standardized positions (so as to cover the whole section) for each slide. PanINs were counted from eight independent animals for each group. Error bars in the figures represent SEM.

#### Model of aggressive PDAC and chemotherapy treatment

For survival studies, we used *Ptf1a*^+/*Cre*^;*Kras*^+/*LSL-G12D*^;*Trp53*^*loxP/loxP*^ (*Kras;p53*) mice, which develop aggressive, quickly fatal disease. *Kras;p53* and *Ptf1a*^+/*Cre*^;*Kras*^+/*LSL-G12D*^;*Trp53*^*loxP/loxP*^;*Smyd2*^*loxP/loxP*^ (*Kras;p53;Smyd2*) mice were followed for signs of disease progression or analyzed at 6 wk of age. At the end point, tumors were processed for biochemical, histological, and immunohistochemical analysis.

#### Model of aggressive PDAC—gemcitabine treatment

To study the sensitivity of PDAC to standard of care chemotherapeutic gemcitabine (Gemzar), *Kras;p53* and *Kras;p53;Smyd2* mutant mice at 40 d of age with fully developed PDAC were treated as indicated with gemcitabine (Sigma-Aldrich) dissolved in saline and were dosed with 100 mg/kg every third day for three cycles as previously described (Olive et al. 2009; Jameson et al. 2013) or vehicle (saline). At the end point, tumors were processed for biochemical, histological, and immunohistochemical analysis.

### Lung cancer mouse models

*Kras*^+/*LSL-G12D*^;*Trp53*^*loxP/loxP*^ (*Kras;p53*) mice were treated with 5 × 10^6^ plaque-forming units (pfu) of adenovirus expressing Cre (University of Iowa Adenovirus Core) by intratracheal infection as previously described (Mazur et al. 2014).

### Histology and immunohistochemistry

Tissue specimens were fixed in 4% buffered formalin for 24 h and stored in 70% ethanol until paraffin-embedding. Three-micrometer sections were stained with hematoxylin and eosin (HE) or Sirius Red or were used for immunohistochemical studies.

Immunohistochemistry was performed on formalin-fixed, paraffin-embedded mouse and human tissue sections using a biotin–avidin method as described before (Mazur et al. 2010). The following antibodies were used at the indicated dilutions: amylase (1:5000; Sigma-Aldrich, no. WH0000276M4), αSMA (1:500; Abcam, no. 5694), CK19 (1:500; Developmental Studies Hybridoma Bank, TromaIII), cleaved Caspase3 (1:200; Cell Signaling, no. 9664), CD45 (1:100; Abcam, no. ab10558), glucagon (1:200; Cell Signaling, no. 8233), insulin (1:500; Cell Signaling, no. 3014), Ki67 (1:1000; BD Bioscience, no. 550609), MUC5AC (1:500; NeoMarkers, no. 145P1), pERK1/2 (1:500; Cell Signaling, no. 4370), pH3 (1:1000; Millipore, no. 06-570), and SMYD2 (1:200; Sigma-Aldrich, no. HPA029023). Sections were developed with DAB and counterstained with hematoxylin. Pictures were taken using a Zeiss microscope equipped with Axiovision software. Analysis of the tumor area and immunohistochemical analysis was done using ImageJ software by measuring pixel units. Human pancreatic and lung adenocarcinoma samples were obtained from deidentified surgical specimens and confirmed by a certified pathologist (E.B. Kaznowska).

### Serum analysis

Cytokine concentrations in serum were measured using the mouse inflammatory cytokine and chemokine Multi-Analyte ELISArray kit (Qiagen) according to the manufacturer's specifications. Data are presented as relative to the wild-type and treatment controls. Sera were collected from the blood samples of individual mice at the end point of the experiment under terminal anesthesia following a protocol for cardiac puncture. Serum samples were separated from blood within 1 h following blood collection by centrifugation at 500*g* for 10 min.

### Preparation of pancreatic epithelial explants culture

Pancreatic epithelial explants from 4- to 6-wk-old *Smyd2* knockout or wild-type mice were established by modification of previously published protocols (Heid et al. 2011; Mazur et al. 2014). In brief, the whole pancreas was harvested and treated twice with 1.2 mg/mL collagenase-VIII (Sigma-Aldrich). Following multiple wash steps with McCoy's medium containing 0.2 mg/mL soybean trypsin inhibitor (SBTI), digested samples were filtered through a 100-μm filter, resuspended in culture medium (Waymouth's MB 752/1 supplemented with 0.1% BSA, 0.2 mg/mL SBTI, 50 µg/mL bovine pituitary extract, 10 µg/mL insulin, 5 µg/mL transferrin, 6.7 ng/mL selenium in 30% fetal calf serum [FCS]), and allowed to recover for 1 h at 37°C. Thereafter, cells were pelleted and resuspended in culture medium supplemented with 1000 U/mL penicillin G, 100 μg/mL streptomycin, amphotericin B, 0.1% FCS, and an equal volume of rat tail collagen and immediately plated on plates precoated with 2.5 mg/mL of rat tail collagen type I. In stimulation experiments, recombinant human EGF (rhEGF) (Invitrogen) was added at a final concentration of 25 ng/mL. For quantification, acinar explants were seeded in triplicates. Cell clusters were counted from at least three optical fields per well and are reported as a percentage of acinar clusters and duct-like spheres. The quantification was performed in four independent biological replicas with three technical replicas each.

### Cell culture, reagents, and transfections

The authenticated cancer cells—293T, CFPac1, H358, H441, MIA PaCa2, Panc1, PaCa3, and SW1990—were obtained from the American Type Culture Collection and cultured in RPMI medium supplemented with 10% FCS (Life Technologies), 100 U/mL penicillin/streptomycin, and glutamine (Life Technologies). Primary mouse pancreatic cancer lines were obtained from *Kras;p53* and *Kras;p53;Smyd2* mouse PDAC. Cells were grown in Dulbecco's modified Eagle's medium (Life Technologies) supplemented with 10% FCS (Life Technologies), 100 U/mL penicillin/streptomycin, and glutamine (Life Technologies). All cells were cultured at 37°C in a humidified incubator with 5% CO_2_. All cell lines were routinely evaluated for mycoplasma contamination.

### Plasmids, lentiviral cDNA, and shRNA constructs

Bacterial expression plasmids were created using pGEX-6P1 vector. Transient mammalian expression plasmids were created using pCagFlag vector. The different inserts were amplified by PCR using either cDNA or specific clones from the human ORFeome library as a template. Single point mutations of SMYD2 and MAPKAPK3 were generated using the QuikChange site-directed mutagenesis protocol (Stratagene), and clones were confirmed by DNA sequencing. SMYD2 and MAPKAPK3 shRNA targeting untranslated regions (UTRs) were cloned in a pSICOR vector carrying a puromycin resistance gene.

The human SMYD2 shRNA sequence directed against the 3′ UTR was TGTCTGAATCTTGAACTTTATTCAAGAGATAAAGTTCAAGATTCAGACTTTTTTC, and the human MAPKAPK3 shRNA sequence directed against the 3′ UTR was TGCTAAGTGGCTTCCCATTATTCAAGAGATAATGGGAAGCCACTTAGCTTTTTTC. Controls cells were stably infected with the empty vector.

SMYD2 and MAPKAPK3 stable reconstitution plasmids were created using the Gateway cloning system according to the manufacturer's instructions (Invitrogen) with either the wild-type or point mutant constructs into pMSCV-Flag vectors (hygromycin resistance).

### Cell extracts, immunoblot analysis, and immunoprecipitation

For total cell extracts, cells were lysed in RIPA buffer (10 mM Tris-HCl at pH 8, 150 mM NaCl, 1 mM EDTA, 0.5 mM EGTA, 1% Triton, 0.1% SDS, 1 mM PMSF, protease inhibitors [Roche], a phosphatase inhibitor cocktail [Sigma-Aldrich]) for 15 min. Cell fractionation was performed by collecting supernatant (cytoplasmic fraction) after centrifugation at 1300*g* for 5 min following a 10-min incubation in hypotonic buffer (10mM HEPES at pH 7.9, 10 mM KCl, 1.5 mM MgCl_2_, 0.34 M sucrose, 10% glycerol, 1 mM DTT, 0.05% Triton, protease inhibitors). The pellet was then incubated for 15 min in LSDB250 buffer (20% glycerol, 3 mM MgCl_2_, 50 mM HEPES at pH 7.9, 250 mM KCl, 0.5 mM DTT, 0.5 mM PMSF, 0.1% NP40, protease inhibitors) and centrifuged at 15,000*g* for 10 min. The supernatant was collected as soluble nuclear extract, and the pellet was further extracted in LSDB250 buffer with sonication (chromatin fraction). Protein concentration was determined by the BCA assay (Pierce).

For immunoprecipitation, cells were lysed in hypotonic buffer for cytoplasmic extract, and the same amount of protein extracts was incubated with specific antibody overnight at 4°C. Extracts were then incubated with protein A Sepharose beads (GE Healthcare) for 2 h at 4°C.

Proteins were resolved by SDS-PAGE, transferred to nitrocellulose membrane, and analyzed by immunoblot. The following antibodies were used at the indicated dilutions: SMYD2 (1:1000, Cell Signaling; no. 9374), ERK1/2 (1:1000; Cell Signaling, no. 4370), pERK1/2 (1:1000; Cell Signaling, no. 4695), IL1β (1:1000; Cell Signaling, no. 12242), IL6 (1:1000; Cell Signaling, no. 12912), MAPKAPK3 (1:1000; for immunoblot: Cell Signaling, no. 7421; for immunoprecipitation: Abcam, no. ab183040), H3 (1:5000; house-made), LSD1 (1:1000; Cell Signaling, no. 2139), β-Tubulin (1:10,000; Millipore, no. 05-661), and β-Actin (1:5000; Sigma-Aldrich, no. A5316). For the MAPKAPK3-K355me1 antibody, we screened house-made or commercially available anti-lysine methyl antibodies that, based on peptide array analyses, might have MAPKAPK3 anti-methyl activity. In total, we tested 53 antibodies for specific detection of MAPKAPK3K355me1 peptide versus MAPKAPK3K355me0 peptide. Of the 53 antibodies screened, we identified 12 potential positives. These were further screened for methyl state specificity using recombinant full-length MAPKAPK3 in vitro methylated by SMYYD2 (or SMYD2 catalytic dead as control). We identified one specific MAPKAPK3K355me1 antibody, originally designed as H3K79me1 (Abcam, no. ab2886, lot GR64011-2). In our assays, this antibody did not detect H3K79me1.

### Cell assays

#### Cell viability

Cells were seeded in 96-well plates at 2000 cells per well (optimum density for growth) in a total volume of 100 μL of medium containing 2% fetal bovine serum. Serially diluted compounds in 100 μL of medium were added to the cells 12 h later. After 72 h of incubation, cell viability was assessed by an MTT assay (Roche) according to the manufacturer's instructions.

#### Colony formation assay

For long-term colony formation assay, 10,000–50,000 cells per well were seeded in six-well plates and treated as indicated. After 9 d, cells were fixed with methanol, stained with Sapphire 700 (LiCor), and imaged (Odyssey Imager, LiCor).

### Xenograft studies

For xenograft analysis, 1 × 10^6^ SW1990 cells were implanted subcutaneously into the flanks of NSG mice with Matrigel (BD Bioscience). Tumor volumes were determined using digital calipers using the formula (length × width × width)/2. Xenograft tumors were treated with 50 mg/kg SMYD2 inhibitor BAY598 once daily (http://www.thesgc.org/chemical-probes/BAY-598), 100 mg/kg gemcitabine every third day (Sigma-Aldrich), or 10% vehicle (2-hydroxypropyl)-β-cyclodextrin (Sigma-Aldrich) via intraperitoneal injection.

### Expression and purification of recombinant proteins

For expression of GST-tagged recombinant proteins, transformed BL21 cells were induced with 0.1 mM IPTG overnight at 20°C, and proteins were purified using glutathione Sepharose beads (GE Healthcare) and eluted in 10 mM reduced glutathione (Sigma) or cleaved from the GST tag using purified Precision enzyme.

### ProtoArray, methylation assays, and MS analysis

In vitro methylation assays were performed using 1–2 µg of recombinant protein substrates incubated with 1 µg of recombinant methyltransferases and 0.1 mM SAM (Sigma), 0.1 mM S-adenosyl-l-methionine-d3 tetra (p-toluenesulfonate) salt (deuterated SAM, CDN isotope) or 2 µCi of ^3^H-AdoMet (American Radiolabeled Chemicals) in buffer containing 50 mM Tris-HCl (pH 8.0), 10% glycerol, 20 mM KCl, 5 mM MgCl_2_, and 1 mM PMSF overnight at 30°C. The reaction mixture was resolved by SDS-PAGE followed by autoradiography, Coomassie stain (Pierce), or MS analysis. Similar conditions were used for methylation assays on ProtoArray (Invitrogen), except that 50 µg of recombinant SMYD2 was incubated on the arrays with 25 µCi of ^3^H-AdoMet (American Radiolabelled Chemicals) overnight at 30°C and analyzed by autoradiography and GenePix software (Molecular Devices). For SMYD2 inhibition, 10 µL of BAY-598 or DMSO was first incubated with recombinant SMYD2 in methylation buffer reaction for 1 h at 30°C, and then 2 µCi of 3H-AdoMet was added to the mix and incubated overnight at 30°C. The selective SMYD2 inhibitor BAY-598 was obtained from the Structural Genomics Consortium (SGC; http://www.thesgc.org/chemical-probes/BAY-598).

For LC-MS/MS analysis of recombinant MAPKAPK3 methylation, deuterated SAM was used to rule out possible artifactual chemical methylation in vitro, shifting the mass of one methyl group from 14.016 Da to 17.034 Da. For Flag immunoprecipitation of MAPKAPK3, SMYD2-HA and MAPKAPK3-Flag were transiently expressed in HEK293T cells for 36 h. After SDS-PAGE separation and Coomassie (GelCode Blue, Thermo) or silver staining (SilverQuest silver staining kit, Invitrogen) according to the manufacturers’ instructions, recombinant or immunoprecipitated MAPKAPK3 was sliced from gels and treated or not with propionylate lysine prior to trypsin digestion (Promega) to increase peptide recovery. Peptides were desalted using C18 stage tips (Thermo Scientific). Peptides were separated by high-performance liquid chromatography (HPLC) using an Ekspert NanoLC 420 (AB Sciex) and analyzed with an Orbitrap Elite mass spectrometer (Thermo Scientific). Data were analyzed using MaxQuant version 1.3.0.5 with a 1% false discovery rate (FDR) for proteins and peptides and allowing methionine oxidation, acetylation of protein N termini, and monomethylation, dimethylation, and trimethylation of lysine as variable modifications. Candidate methylation sites were verified by manual inspection.

### Meta-analysis of public PDAC data sets

We downloaded raw data for eight publically available PDAC gene expression studies from the NCBI Gene Expression Omnibuse and European Bioinformatics Institute ArrayExpress. After reannotating the probes, each data set was separately normalized using GC robust multiarray average (GCRMA). We applied two meta-analysis approaches to the normalized data (Khatri et al. 2013). Briefly, the first approach combined the effect sizes from each data set into a meta-effect size to estimate the amount of changes in expression across all data sets. For each gene in each data set, an effect size was computed using Hedges’ adjusted *g*. If multiple probes mapped to a gene, the effect size for each gene was summarized using the fixed effect inverse variance model. We combined study-specific effect sizes to obtain the pooled effect size and its standard error using the random effects inverse variance technique. We computed *z* statistics as a ratio of the pooled effect size to its standard error for each gene and compared the result with a standard normal distribution to obtain a nominal *P*-value. *P*-values were corrected for multiple hypotheses testing using FDR. We used a second nonparametric meta-analysis that combines *P*-values from individual experiments to identify genes with a large effect size in all data sets. Briefly, we calculated a *t* statistic for each gene in each study. After one-tail *P*-values for each gene were computed, they were corrected for multiple hypotheses using FDR. Next, we used Fisher's sum of logs method, which sums the logarithm of corrected *P*-values across all data sets for each gene and compares the sum against a χ^2^ distribution with 2*k* degrees of freedom, where *k* is the number of data sets used in the analysis.

### Statistics

Kaplan-Meier survival curves were calculated using the survival time for each mouse from all littermate groups. The log-rank test was used to test for significant differences between the groups. For image quantification and gene expression analysis, statistical significance was assayed by Student's *t*-test with Prism GraphPad software (two-tailed unpaired and paired *t*-test depending on the experiment; variance was first systematically examined using an *F*-test). In the figures, *P* < 0.05 is indicated by an asterisk, *P* < 0.01 is indicated by two asterisks, *P* < 0.001 is indicated by three asterisks, and not significant is indicated by ns. Data are represented as mean ± standard error of the mean (SEM).

### Competing interest statement

O.G. is a cofounder of EpiCypher, Inc. T.S and C.S. are employees of Bayer Pharma AG, and their contribution to this research work was conducted under the employment of Bayer Pharma AG.

## Supplementary Material

Supplemental Material
