# Supplementary material for: Coordination of stress signals by the lysine methyltransferase SMYD2 promotes pancreatic cancer
Source: Genes Dev. 2016 Apr 1;30(7):772–85. doi: 10.1101/gad.275529.115 (PMC4826394; doi:10.1101/gad.275529.115)
Supplement: Supplemental Material [file supp_30_7_772__index.html]

Coordination of stress signals by the lysine methyltransferase SMYD2 promotes pancreatic cancer — Supplemental Material 

# Coordination of stress signals by the lysine methyltransferase SMYD2 promotes pancreatic cancer

## Supplemental Material

**Files in this Data Supplement:**

- Supp Figures.pdf
- Supp Table S1.pdf
- Supp Text.docx
