# Supplementary material for: Coordination of stress signals by the lysine methyltransferase SMYD2 promotes pancreatic cancer
Source: Genes Dev. 2016 Apr 1;30(7):772–85. doi: 10.1101/gad.275529.115 (PMC4826394; doi:10.1101/gad.275529.115)
Supplement: Supplemental Material [file supp_gad.275529.115_Supp_Figures.pdf]

Reynoird_Supplemental_FigS1

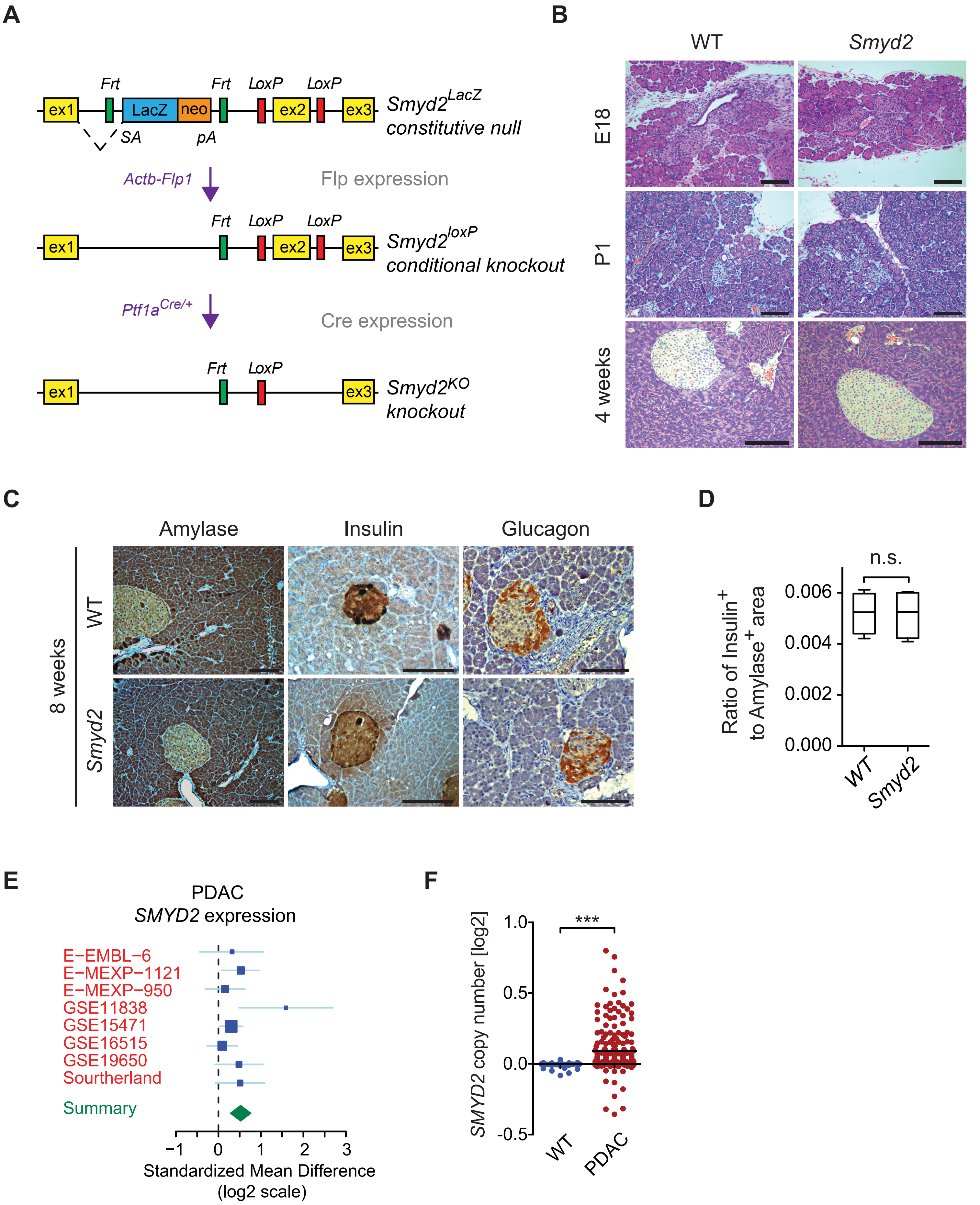

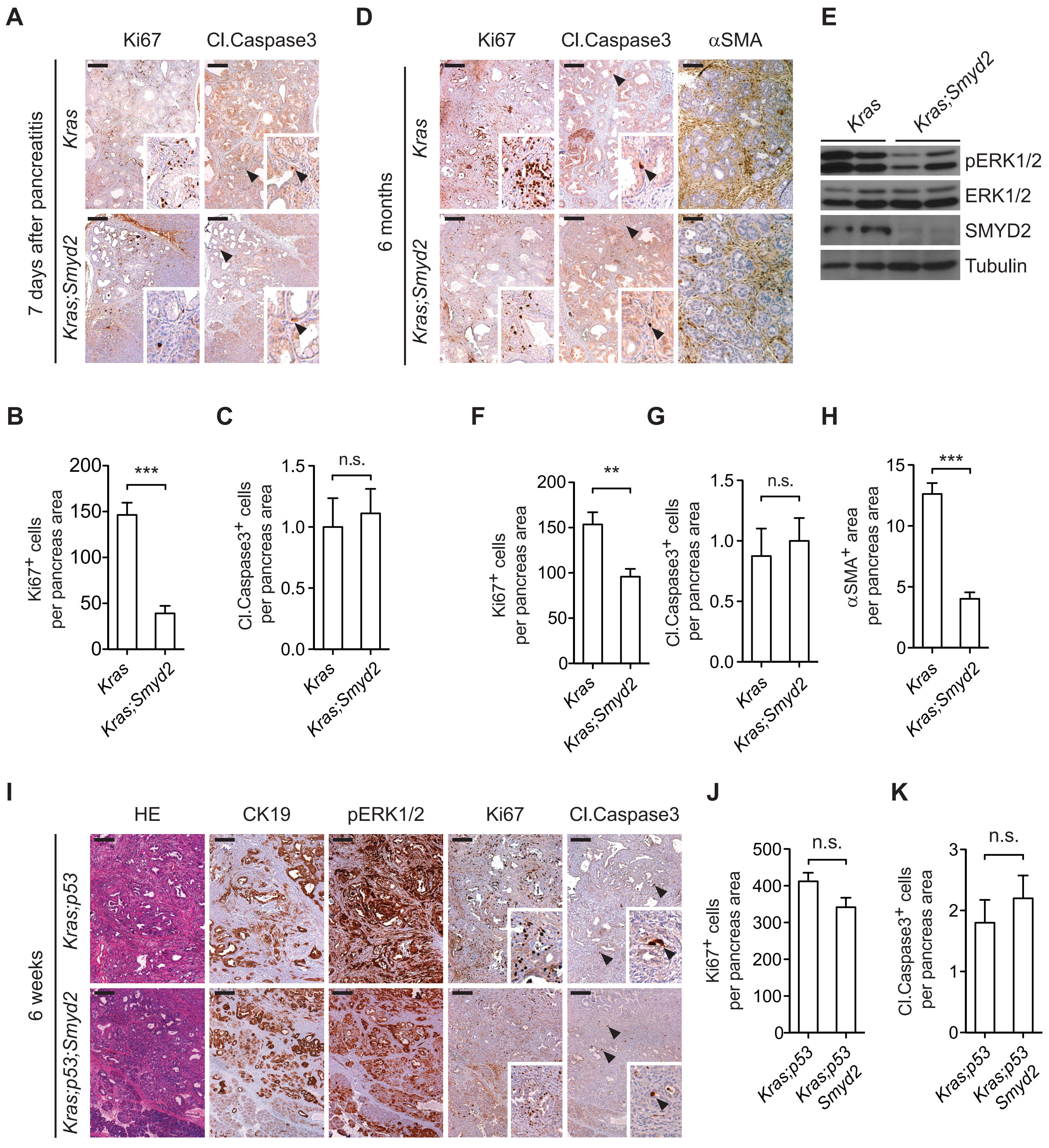
A
Ki67
Cl.Caspase3
7 days after pancreatitis
Kras
Kras;Smyd2
D
Ki67
Cl.Caspase3
αSMA
6 months
Kras
Kras;Smyd2
E
Kras
Kras;Smyd2
pERK1/2
ERK1/2
SMYD2
Tubulin
B
Ki67+ cells per pancreas area
***
200
150
100
50
0
Kras
Kras;Smyd2
C
Cl.Caspase3+ cells per pancreas area
n.s.
1.5
1.0
0.5
0.0
Kras
Kras;Smyd2
F
Ki67+ cells per pancreas area
**
200
150
100
50
0
Kras
Kras;Smyd2
G
Cl.Caspase3+ cells per pancreas area
n.s.
1.5
1.0
0.5
0.0
Kras
Kras;Smyd2
H
αSMA+ area per pancreas area
***
15
10
5
0
Kras
Kras;Smyd2
I
HE
CK19
pERK1/2
Ki67
Cl.Caspase3
6 weeks
Kras;p53
Kras;p53;Smyd2
J
Ki67+ cells per pancreas area
n.s.
500
400
300
200
100
0
Kras;p53
Kras;p53 Smyd2
K
Cl.Caspase3+ cells per pancreas area
n.s.
3
2
1
0
Kras;p53
Kras;p53 Smyd2

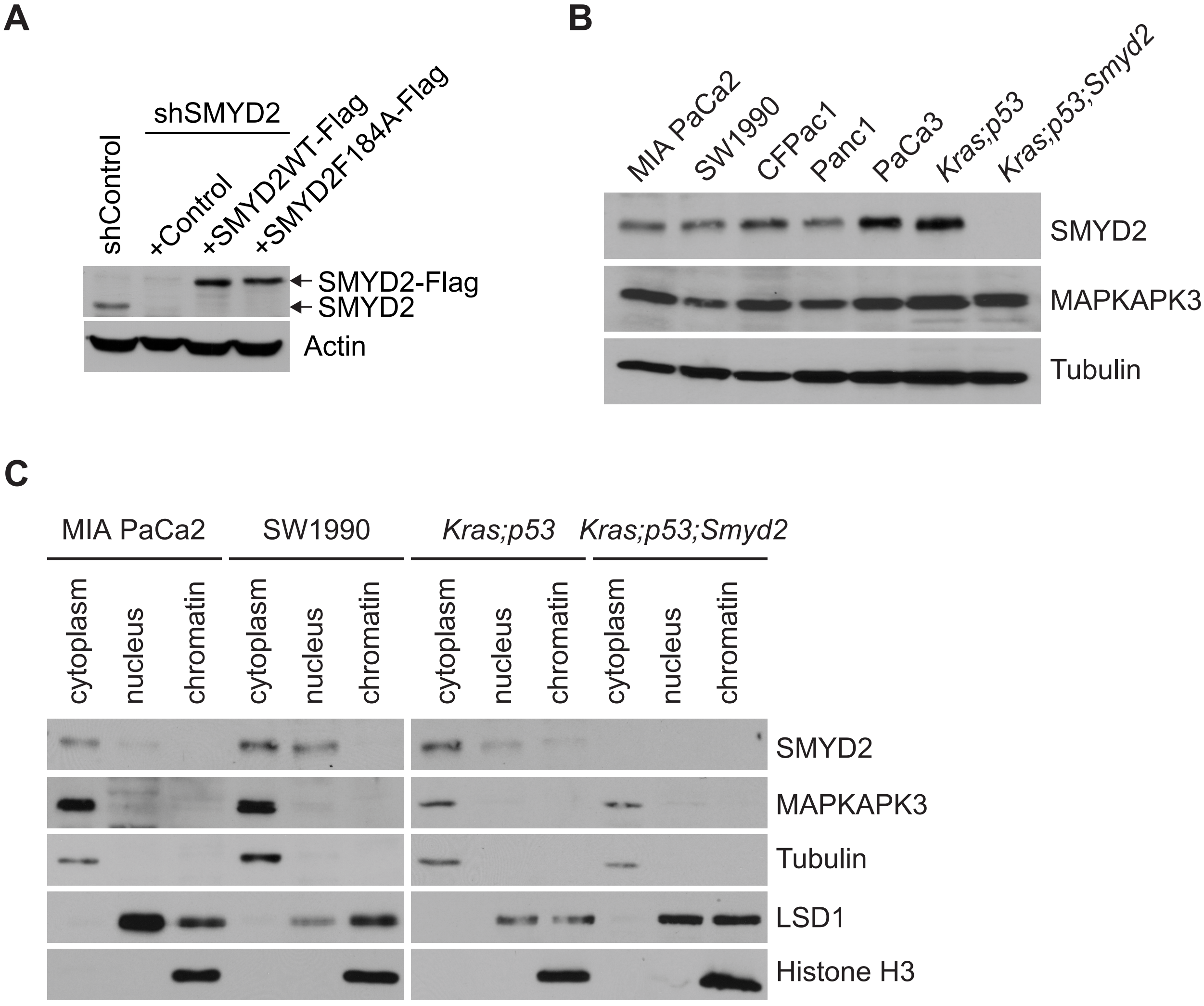
A
shSMYD2
shControl
+Control
+SMYD2WT-Flag
+SMYD2F184A-Flag
SMYD2-Flag
SMYD2
Actin
B
MIA PaCa2
SW1990
CFPac1
Panc1
PaCa3
Kras;p53
Kras;p53;Smyd2
SMYD2
MAPKAPK3
Tubulin
C
MIA PaCa2
SW1990
Kras;p53
Kras;p53;Smyd2
cytoplasm
nucleus
chromatin
SMYD2
MAPKAPK3
Tubulin
LSD1
Histone H3

Reynoird_Supplemental_FigS4

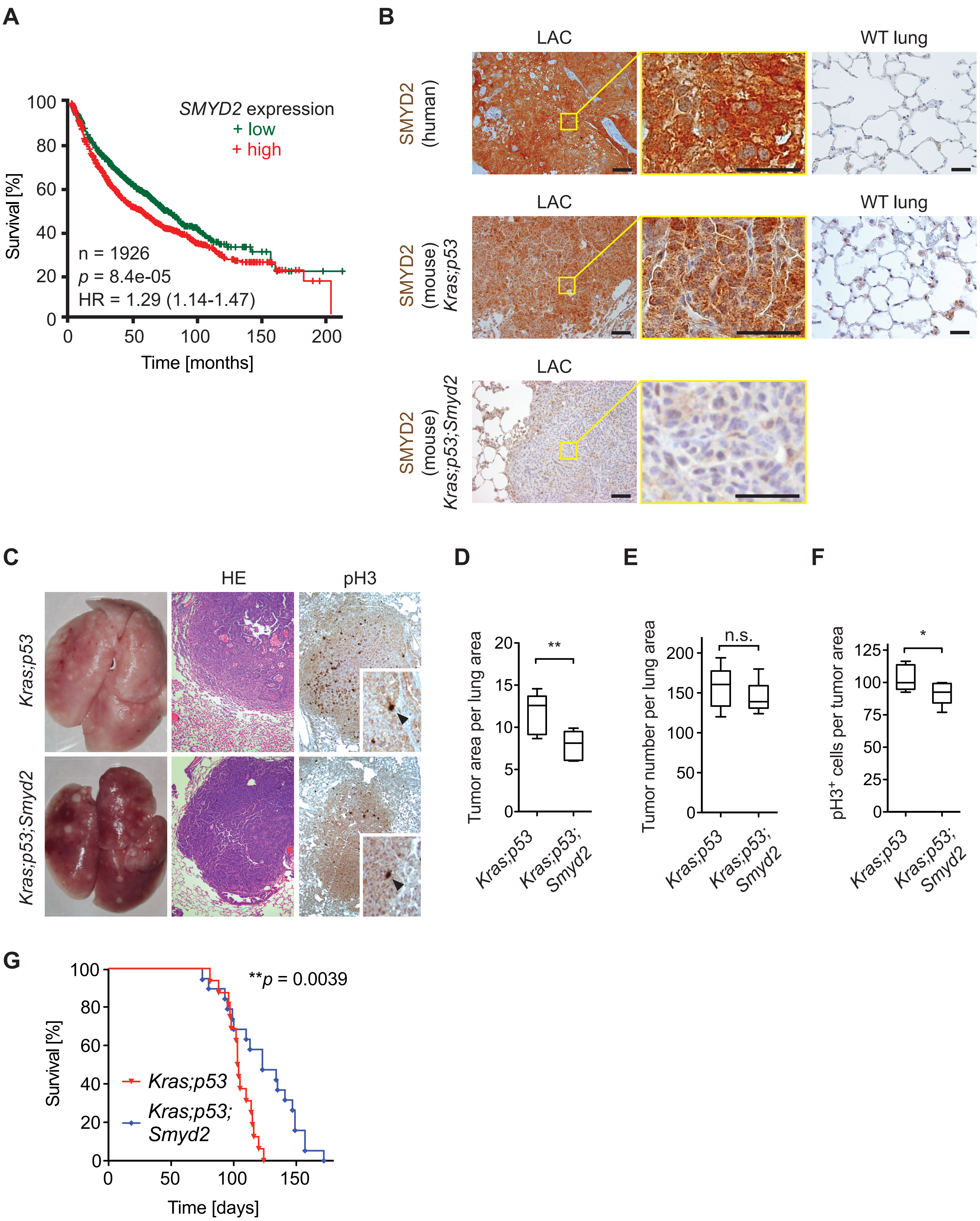

Reynoird_Supplemental_FigS5

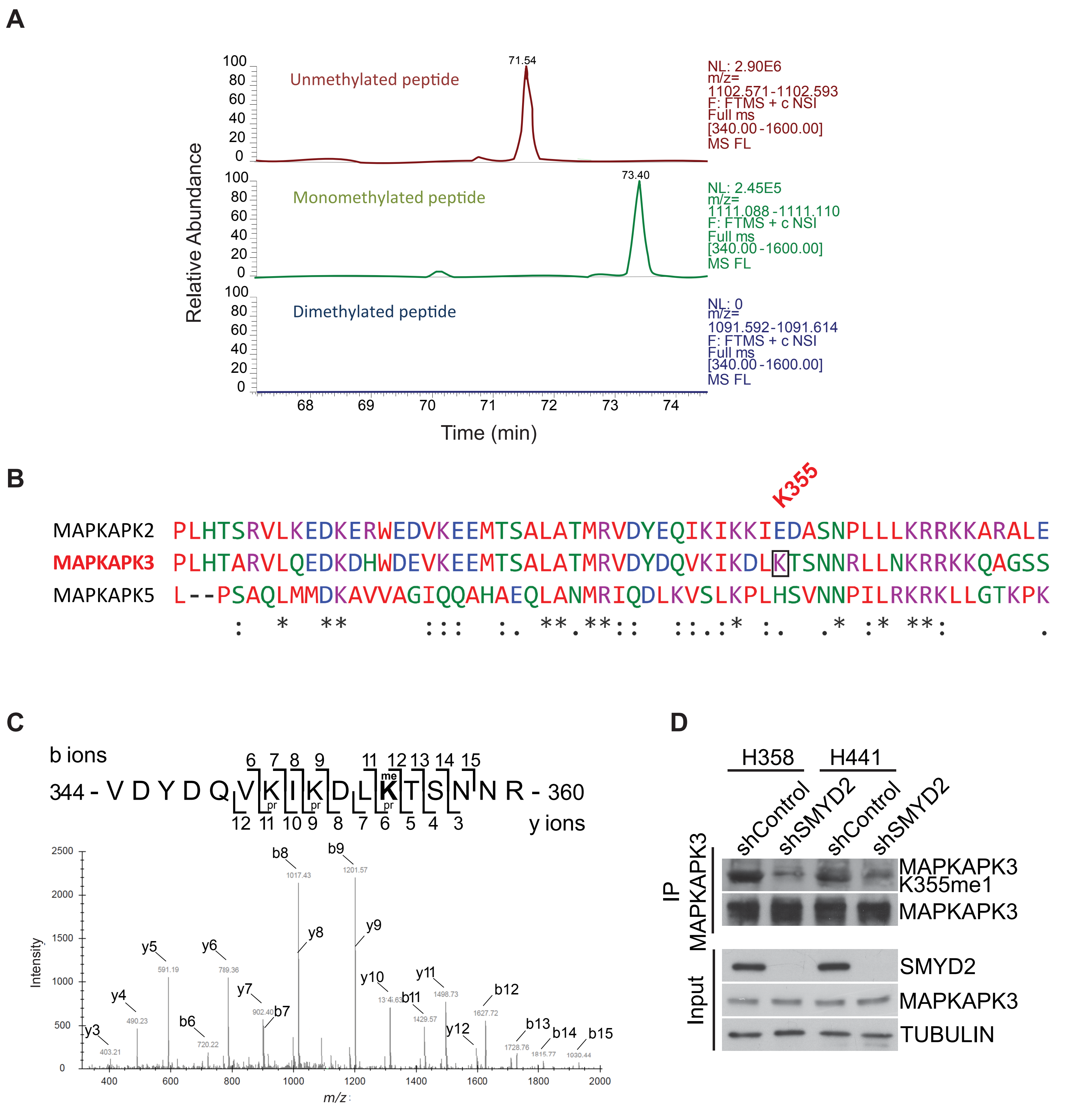

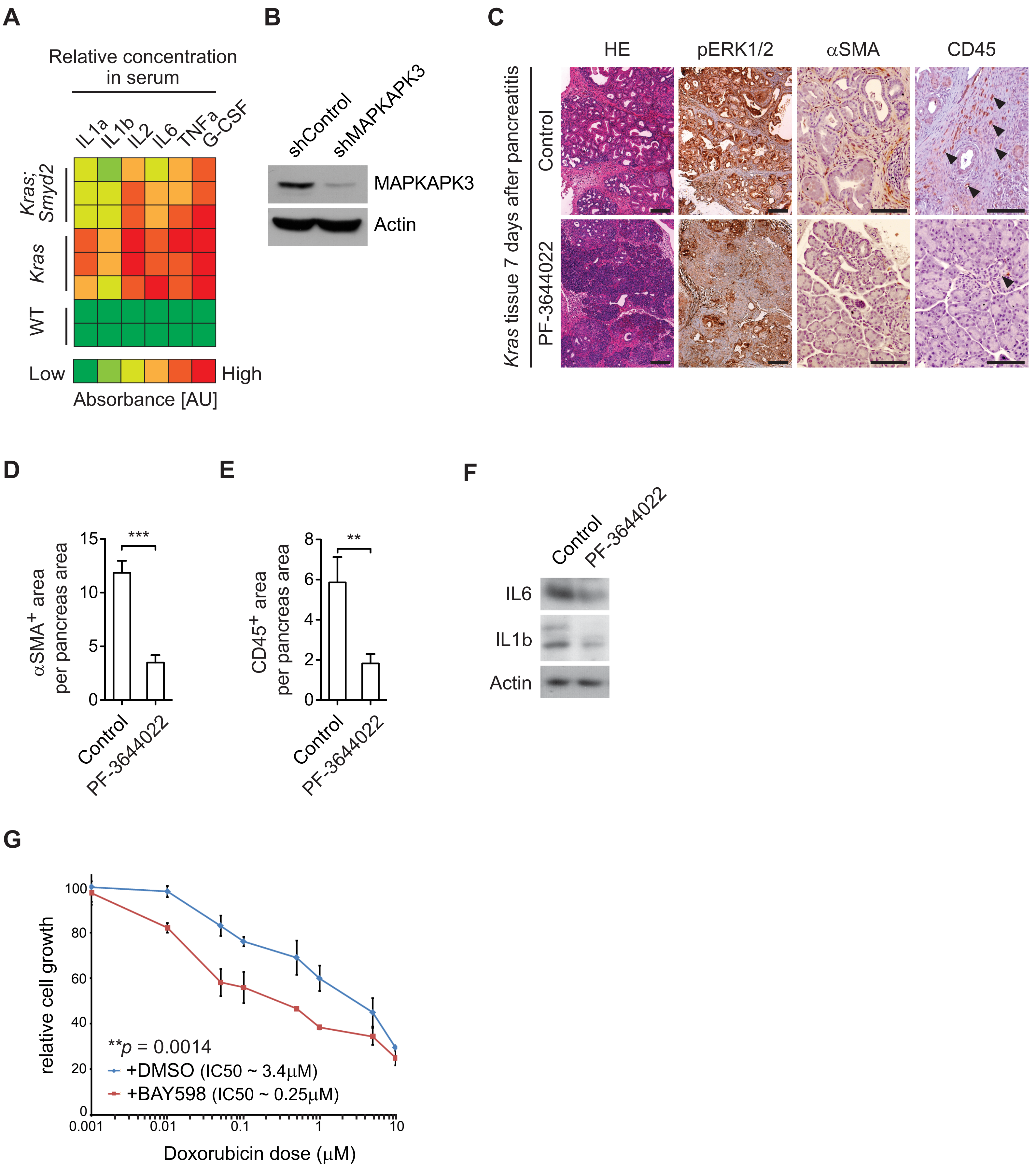
A
Relative concentration in serum
IL1a IL1b IL2 IL6 TNFa G-CSF
Kras; Smyd2
Kras
WT
Low
High
Absorbance [AU]
B
shControl
shMAPKAPK3
MAPKAPK3
Actin
C
HE
pERK1/2
αSMA
CD45
Kras tissue 7 days after pancreatitis
Control
PF-3644022
D
αSMA+ area per pancreas area
***
15
10
5
0
Control
PF-3644022
E
CD45+ area per pancreas area
**
8
6
4
2
0
Control
PF-3644022
F
Control
PF-3644022
IL6
IL1b
Actin
G
relative cell growth
100
80
60
40
20
0
**p = 0.0014
+DMSO (IC50 ~ 3.4μM)
+BAY598 (IC50 ~ 0.25μM)
0.001
0.01
0.1
1
10
Doxorubicin dose (μM)
