# Supplementary material for: Coordination of stress signals by the lysine methyltransferase SMYD2 promotes pancreatic cancer
Source: Genes Dev. 2016 Apr 1;30(7):772–85. doi: 10.1101/gad.275529.115 (PMC4826394; doi:10.1101/gad.275529.115)
Supplement: Supplemental Material [file supp_gad.275529.115_Supp_TableS1.pdf]

Reynoird_Supplemental Table S1

List of potential SMYD2 substrate candidates detected on two independent radiolabeled protoarray methylation assays.
Each protein is spotted twice as a doublet on the protoarray. To be identified as a methylated candidate, the protein doublet has to be radiolabeled in both protoarrays. The internal replicates represent protein doublet spotted more than once on each individual protoarray.

	hits in both protoarrays	hits in protoarray 1	internal replicates protoarray 1	hits in protoarray 2	internal replicates protoarray 2
ADH4	X	X	1/1	X	1/1
AGGF1	X	X	1/1	X	1/1
AHI1	X	X	1/1	X	1/1
ALK	X	X	1/1	X	1/1
AMMECR1L	X	X	1/1	X	1/1
ANKZF1	X	X	1/1	X	1/1
APEX1	X	X	1/1	X	1/1
APOBEC3C	X	X	1/1	X	1/1
APTX	X	X	1/1	X	1/1
ASAP	X	X	1/1	X	1/1
ASXL1	X	X	1/1	X	1/1
AURKA	X	X	1/1	X	1/1
AURKB	X	X	1/1	X	1/1
BBX	X	X	1/1	X	1/1
BMX	X	X	3/3	X	2/3
BTF3	X	X	1/1	X	1/1
BUD31	X	X	1/1	X	1/1
BYSL	X	X	2/2	X	2/2
C11orf31	X	X	1/1	X	1/1
C14orf111	X	X	1/1	X	1/1
C17orf85	X	X	1/1	X	1/1
C1orf116	X	X	1/1	X	1/1
C1orf55	X	X	1/1	X	1/1
C22orf33	X	X	1/1	X	1/1
C6orf130	X	X	1/1	X	1/1
C6orf166	X	X	1/1	X	1/1
C8orf59	X	X	1/1	X	1/1
C9orf32	X	X	1/1	X	1/1
C9orf9	X	X	1/1	X	1/1
CASK	X	X	1/1	X	1/1
CCDC23	X	X	1/1	X	1/1
CCDC55	X	X	1/1	X	1/1
CCL19	X	X	1/1	X	1/1
CDC7	X	X	1/1	X	1/1
CDK5	X	X	2/2	X	2/2
CDKL1	X	X	1/1	X	1/1
CDKN2AIP	X	X	1/1	X	1/1
CFDP1	X	X	1/1	X	1/1
CORO6	X	X	1/1	X	1/1
CSNK1G2	X	X	1/1	X	1/1
CSNK2A2	X	X	1/1	X	1/1
CT45A1	X	X	1/1	X	1/1
CUTC	X	X	2/2	X	2/2
DAPK3	X	X	1/1	X	1/1
DCUN1D2	X	X	1/1	X	1/1
DDX1	X	X	1/1	X	1/1
DDX21	X	X	1/1	X	1/1
DDX49	X	X	1/1	X	1/1
DDX54	X	X	1/1	X	1/1
DDX6	X	X	1/1	X	1/1
DGCR8	X	X	1/1	X	1/1
DKFZp761G2113	X	X	1/1	X	1/1
DNAJB7	X	X	1/1	X	1/1
DNAJC8	X	X	1/1	X	1/1
DNTT	X	X	1/1	X	1/1
EDF1	X	X	1/1	X	1/1
EFS	X	X	1/1	X	1/1
EHHADH	X	X	1/1	X	1/1
ELOF1	X	X	1/1	X	1/1
EPB41L4A	X	X	1/1	X	1/1
FAM116B	X	X	1/1	X	1/1
FAM128A	X	X	1/1	X	1/1
FAM131B	X	X	1/1	X	1/1
FAU	X	X	1/1	X	1/1

FGD2	X	X	1/1	X	1/1
FGF12	X	X	3/3	X	2/3
FLJ11184	X	X	1/1	X	1/1
FSD1	X	X	1/1	X	1/1
GABARAP	X	X	1/1	X	1/1
GABARAPL2	X	X	1/1	X	1/1
GAS2L3	X	X	1/1	X	1/1
GCC1	X	X	1/1	X	1/1
GLIPR2	X	X	1/1	X	1/1
GTPBP10	X	X	1/1	X	1/1
HIST1H2BD	X	X	1/1	X	1/1
HMGN1	X	X	2/2	X	2/2
HMGN3	X	X	1/1	X	1/1
HNRPA1	X	X	1/1	X	1/1
HP1BP3	X	X	1/1	X	1/1
HSP90AA1	X	X	1/1	X	1/1
IFIT5	X	X	1/1	X	1/1
IL21	X	X	1/1	X	1/1
IMPACT	X	X	1/1	X	1/1
INSR	X	X	1/1	X	1/1
JMJD2D	X	X	1/1	X	1/1
KCTD18	X	X	2/2	X	2/2
KIAA0515	X	X	1/1	X	1/1
KIAA1530	X	X	1/1	X	1/1
KPNA2	X	X	2/2	X	2/2
LOC153684	X	X	1/1	X	1/1
LOC57228	X	X	1/1	X	1/1
LRRC6	X	X	1/1	X	1/1
LSM4	X	X	1/1	X	1/1
MAP2	X	X	1/1	X	1/1
MAPKAPK3	X	X	1/1	X	1/1
MCM10	X	X	1/1	X	1/1
METAP2	X	X	1/1	X	1/1
MPP7	X	X	1/1	X	1/1
MYLK	X	X	1/1	X	1/1
NEK7	X	X	1/1	X	1/1
NKRF	X	X	1/1	X	1/1
NOL7	X	X	1/1	X	1/1
PAK4	X	X	1/1	X	1/1
PDAP1	X	X	1/1	X	1/1
PHF20L1	X	X	1/1	X	1/1
PIN4	X	X	1/1	X	1/1
PLK1	X	X	1/1	X	1/1
PRC1	X	X	1/1	X	1/1
PRKAA2	X	X	1/1	X	1/1
PRKAG2	X	X	1/1	X	1/1
PRKRA	X	X	1/1	X	1/1
PRR15	X	X	1/1	X	1/1
PSMD4	X	X	1/1	X	1/1
PSRC1	X	X	1/1	X	1/1
PTPN6	X	X	1/1	X	1/1
PUS7L	X	X	1/1	X	1/1
RABL3	X	X	1/1	X	1/1
RDBP	X	X	1/1	X	1/1
RECQL	X	X	1/1	X	1/1
RIT1	X	X	1/1	X	1/1
RIT2	X	X	2/2	X	2/2
RP13	X	X	1/1	X	1/1
RPL22	X	X	1/1	X	1/1
RPL23A	X	X	1/1	X	1/1
RPL29	X	X	1/1	X	1/1
RPS25	X	X	1/1	X	1/1
RPS27L	X	X	1/1	X	1/1
RPS28	X	X	1/1	X	1/1
RSBN1L	X	X	1/1	X	1/1
RTF1	X	X	1/1	X	1/1
RTN4	X	X	1/1	X	1/1
SCEL	X	X	1/1	X	1/1
SELS	X	X	1/1	X	1/1
SERBP1	X	X	1/1	X	1/1
SFRS1	X	X	1/1	X	1/1
SFRS2	X	X	1/1	X	1/1
SFRS5	X	X	1/1	X	1/1
SNRPA1	X	X	2/2	X	2/2
SPANXN4	X	X	1/1	X	1/1

SPRR4	X	X	1/1	X	1/1
SRP19	X	X	2/2	X	1/2
STK6	X	X	1/1	X	1/1
SUDS3	X	X	1/1	X	1/1
SURF2	X	X	1/1	X	1/1
SYTL2	X	X	1/1	X	1/1
TARBP2	X	X	1/1	X	1/1
TBC1D22B	X	X	1/1	X	1/1
TCEA1	X	X	1/1	X	1/1
TCEAL1	X	X	1/1	X	1/1
TP53I13	X	X	1/1	X	1/1
TTK	X	X	1/1	X	1/1
UBD	X	X	2/2	X	2/2
UBE2E2	X	X	1/1	X	1/1
UBE2S	X	X	1/1	X	1/1
UBXD3	X	X	1/1	X	1/1
UTP14A	X	X	1/1	X	1/1
VAMP4	X	X	2/2	X	2/2
VRK1	X	X	1/1	X	1/1
WDR5	X	X	1/1	X	1/1
6CKINE			0/1	X	1/1
ABCF1		X	1/1		0/1
ADAM22		X	1/1		0/1
ADD2			0/1	X	1/1
AFF4		X	1/1		1/1
ALKBH3		X	1/1		0/1
ANK1		X	1/1		0/1
ARHGEF5			0/1	X	1/1
ARL6IP4			0/1	X	1/1
ARPC1B			0/1	X	1/1
ATP6AP2		X	1/1		0/1
BAZ2B			0/1	X	1/1
BCKDK			0/1	X	1/1
BCL7B			0/1	X	1/1
BCL7C			0/1	X	1/1
BRDG1			0/1	X	1/1
C10orf63		X	1/1		0/1
C11orf57		X	1/1		0/1
C11orf75		X	1/1		0/1
C12orf31		X	1/1		0/1
C12orf52		X	1/1		0/1
C14orf106		X	1/1		0/1
C14orf111			0/1	X	1/1
C16orf78		X	1/1		0/1
C17orf64		X	1/1		0/1
C19orf43		X	1/1		0/1
C1orf174		X	1/1		0/1
C1orf35		X	1/1		0/1
C1orf64		X	1/1		0/1
C21orf7		X	1/1		0/1
C6orf201			0/1	X	1/1
C8orf70		X	1/1		0/1
C9orf19		X	1/1		0/1
CAMK2B		X	1/1		0/1
CBX5		X	1/1		0/1
CCDC28A		X	1/1		0/1
CCDC5		X	1/1		0/1
CCDC72		X	1/1		0/1
CCDC75		X	1/1		0/1
CCNB3		X	1/1		0/1
CDC2			0/1	X	1/1
CDCA3		X	1/1		0/1
CDKL3			0/1	X	1/1
CENPQ		X	1/1		0/1
CGI			0/1	X	1/1
CHEK2			0/1	X	1/1
CHMP2B			0/1	X	1/1
CKAP2		X	1/1		0/1
CLIC5			0/1	X	1/1
COPS5			0/1	X	1/1
CSAG1		X	1/1		0/1
CSAG3A			0/1	X	1/1
CSF1R		X	1/1		0/1
CSNK1G1			0/1	X	1/1
CSNK2A1		X	2/2		0/2

CTNND1			0/1	X	1/1
CUEDC1		X	1/1		0/1
CXorf56		X	1/1		0/1
DCX			0/1	X	1/1
DCX			0/1	X	1/1
DDX55			0/1	X	1/1
DIMT1L		X	1/1		0/1
DNALI1		X	1/1		0/1
EDN3		X	1/1		0/1
EIF1AX		X	1/1		0/1
EIF1AY			0/1	X	1/1
EIF2A			0/1	X	1/1
EIF2S2		X	1/1		0/1
EPHA3			0/1	X	1/1
FAM122A		X	1/1		0/1
FAM128B		X	1/1		0/1
FAM130A2			0/1	X	1/1
FAM21C			0/1	X	1/1
FAM50A		X	1/1		0/1
FAM54A			0/1	X	1/1
FEN1		X	1/1		0/1
FGF16		X	1/1		0/1
FGFR1OP		X	1/1		0/1
FKBP3		X	1/1		0/1
GABARAPL1			0/1	X	1/1
GMNN		X	1/1		0/1
GNL2		X	1/1		0/1
GOSR1		X	1/1		0/1
GSK3B		X	1/1		0/1
GTF2E2		X	1/1		0/1
HADH			0/1	X	1/1
HDGFRP3			0/1	X	1/1
HIST2H2AC		X	1/1		0/1
HMGB2		X	1/1		0/1
HSD3B2		X	1/1		0/1
HSPC111		X	1/1		0/1
HSPC148		X	1/1		0/1
IL20			0/1	X	1/1
IPPK			0/1	X	1/1
ISG20		X	1/1		0/1
ITAC			0/1	X	1/1
KCNAB1			0/1	X	1/1
KCNAB2		X	1/1		0/1
KIAA0020			0/1	X	1/1
KIAA0859			0/1	X	1/1
KIF3A			0/1	X	1/1
KIF3B			0/1	X	1/1
KRR1		X	1/1		0/1
LAD1			0/1	X	1/1
LAMC1			0/1	X	1/1
LARP1			0/1	X	1/1
LENG1		X	1/1		0/1
LETM2		X	1/1		0/1
LIMS2			0/1	X	1/1
LMOD1		X	2/2		0/2
LOC285927		X	1/1		0/1
LOC389415			0/1	X	1/1
LOC401068			0/1	X	1/1
LRRC20		X	1/1		0/1
MAP3K2			0/1	X	1/1
MCM2			0/1	X	1/1
METTL1		X	1/1		0/1
MGC17403		X	1/1		0/1
NCAPG		X	1/1		0/1
NEIL1		X	1/1		0/1
NIP30		X	1/1		0/1
NOLC1		X	1/1		0/1
NOSTRIN			0/1	X	1/1
NUAK1		X	1/1		0/1
NUSAP1		X	1/1		0/1
ODF2			0/1	X	1/1
PCDHGA5		X	1/1		0/1
PCLO			0/1	X	1/1
PCMT1			0/1	X	1/1
PCMT1			0/1	X	1/1

PIP5K2C			0/1	X	1/1
PLEKHA1		X	1/1		0/1
PMP2		X	1/1		0/1
POLB			0/1	X	1/1
PPAN			0/1	X	1/1
PPHLN1		X	1/1		0/1
PRR16			0/1	X	1/1
PRRC1		X	1/1		0/1
PSMG1		X	1/1		0/1
PTCD2		X	1/1		0/1
PTHLH		X	1/1		0/1
PTRH1			0/1	X	1/1
PWWP2B		X	1/1		0/1
RAB35		X	1/1		0/1
RAC1			0/1	X	1/1
RAD51AP1			0/1	X	1/1
RBM34		X	1/1		0/1
RCOR3		X	1/1		0/1
RG9MTD1			0/1	X	1/1
RGS3		X	1/1		0/1
RNF32		X	1/1		0/1
RNF4		X	1/1		0/1
RPL11			0/1	X	1/1
RPL30			0/1	X	1/1
RPL31			0/1	X	1/1
RPL39L			0/1	X	1/1
RPS19			0/1	X	1/1
RPS20			0/1	X	1/1
RSU1		X	1/1		0/1
SFRS2B		X	1/1		0/1
SFRS6			0/1	X	1/1
SFRS9		X	1/1		0/1
SH3YL1		X	1/1		0/1
SIP1		X	1/1		0/1
SIRT6			0/1	X	1/1
SLAIN2			0/1	X	1/1
SMTNL2		X	1/1		0/1
SNAP25		X	1/1		0/1
SPANXB1		X	1/1		0/1
SSH3			0/1	X	1/1
STAT6			0/1	X	1/1
SUHW2		X	1/1		0/1
SUPT16H		X	1/1		0/1
SUPT16H			0/1	X	1/1
TAF1B		X	1/1		0/1
TBPL1		X	1/1		0/1
TBRG1		X	1/1		0/1
THEX1			0/1	X	1/1
TNIK			0/1	X	1/1
TOB2		X	1/1		0/1
TPT1			0/1	X	1/1
TRA2A			0/1	X	1/1
TRAPPC2		X	1/1		0/1
TRIM41			0/1	X	1/1
TRIT1			0/1	X	1/1
TTLL1			0/1	X	1/1
TTLL7		X	1/1		0/1
UFD1L			0/1	X	1/1
UNKL			0/1	X	1/1
VCY			0/1	X	1/1
WHSC1			0/1	X	1/1
WIT1		X	1/1		0/1
XAF1			0/1	X	1/1
YY1		X	1/1		0/1
ZC3H3			0/1	X	1/1
ZMYND11			0/1	X	1/1
ZNF239		X	1/1		0/1
ZNF323			0/1	X	1/1
