# Supplementary material for: Coordination of stress signals by the lysine methyltransferase SMYD2 promotes pancreatic cancer
Source: Genes Dev. 2016 Apr 1;30(7):772–85. doi: 10.1101/gad.275529.115 (PMC4826394; doi:10.1101/gad.275529.115)
Supplement: Supplemental Material [file supp_gad.275529.115_Supp_Text.docx]

**Coordination of stress signals by the lysine methyltransferase SMYD2 promotes pancreatic cancer** – Reynoird et al.

**SUPPLEMENTAL TABLE AND FIGURE LEGENDS**

**Supplemental Figure S1. Generation of *Smyd2* knockout mice and analysis of pancreas phenotypes.**

(**A**) *Smyd2* mouse models. Insertion of a *LacZ* cassette with a strong splice acceptor in intron 2 of the *Smyd2* gene creates a mutant allele (*Smyd2^LacZ^*). Expression of the Flp recombinase removes the LacZ cassette, creating a conditional allele (*Smyd2^loxP^*). Expression of the Cre recombinase (in the pancreas, with *Ptf1a^+/Cre^* mice) deletes *Smyd2* exon 2, resulting in a null allele (*Smyd2^KO^*).

(**B**) *Smyd2* mice develop normal pancreata. Representative hematoxylin and eosin (HE) staining of pancreata from wild-type and *Ptf1a^+/Cre^;Smyd2^loxP/loxP^* (*Smyd2*) mutant embryos (day 18 of development, E18), newborn mice (P1) and adult mice (4 weeks of age), (n = 5 for each experimental group). Scale bars, 100 μm.

(**C**) *Smyd2* mice develop functional pancreata. Analysis of pancreatic markers in wild-type and *Smyd2* mutant mice at 8 weeks of age. Scale bars, 100 μm.

(**D**) Quantification of the Insulin to Amylase ratio in wild-type and *Smyd2* mutant mice at 8 weeks of age (n = 5 for each experimental group). n.s.: not significant (two-tailed unpaired Student’s t-test). Data are represented as mean +/– SEM.

(**E**) *SMYD2* is overexpressed in PDAC. Bioinformatics meta-analysis of *SMYD2* expression levels in eight (n = 294 independent samples) publicly-available human PDAC gene expression studies from NCBI GEO and EBI ArrayExpress. Detailed statistical description in the Methods section.

(**F**) *SMYD2* gene is amplified in PDAC. Analysis of TCGA data for *SMYD2* copy numbers in human PDAC. ***: P < 0.001 (two-tailed unpaired Student’s t-test) (GISTIC Score q-value across the genome not significant).

**Supplemental Figure S2. *Smyd2* deletion suppresses PDAC growth and improves survival in a PDAC mouse model.**

(**A**) Representative IHC images for Ki67, a marker of proliferation, and cleaved Caspase 3 (cl.Caspase3), a marker of apoptosis in pancreatic section from *Kras* and *Kras;Smyd2* mutant mice 7 days after pancreatitis induction (related to Fig. 1D-G). Scale bars, 100 μm.

(**B-C**) Quantification of Ki67 and cleaved Caspase 3 positive cells (n = 5 for each experimental group).

(**D**) Representative IHC images for Ki67, cleaved Caspase 3, and alpha smooth muscle actin (αSMA), a marker of activated myofibroblasts in the tumor stroma in *Kras* and *Kras;Smyd2* mutant mice (6 months of age) (related to Figure 1H-K). Scale bars, 100 μm.

(**E**) Immunoblot analysis of SMYD2 expression, and total and phosphorylated ERK1/2 (pERK1/2) from tumor extracts (as in D). Tubulin serves as a loading control.

(**F-H**) Quantification of Ki67, cleaved Caspase 3, and αSMA positive cells (n = 5 for each experimental group).

(**I**) Representative IHC images for cytokeratin 19 (CK19), a marker of epithelial tumor cells, pERK1/2, a marker of Ras pathway activity, Ki67, and cleaved Caspase 3 in *Kras;p53* and *Kras;p53;Smyd2* mice (6 weeks of age) (related to Fig. 1L-N). Scale bars, 100 μm.

(**J-K**) Quantification of Ki67 and cleaved Caspase 3 positive cells (n = 5 for each experimental group).

n.s.: not significant; *: *p* < 0.05; **: *p* < 0.01; ***: *p* < 0.001; (p value calculated by two-tailed unpaired Student’s t-test; data are represented as mean +/– SEM).

**Supplemental Figure S3. Immunoblot analysis of SMYD2 and MAPKAPK3 in murine and human PDAC cell lines.**

(**A**) Immunoblot analysis of SMYD2 expression from total protein extracts of SW1990 cells after stable transfection with Control or SMYD2 shRNA plasmids and reconstitution with SMYD2WT-Flag or SMYD2F184A-Flag plasmids (related to Fig. 1O). Actin serves as a loading control.

(**B**) *SMYD2* and *MAPKAPK3* are expressed in various pancreatic cell lines. Immunoblot analysis with the indicated antibodies from total cell extracts of different human pancreatic cancer cell lines and primary PDAC cell lines established form *Kras;p53* and *Kras;p53;Smyd2* mutant mice. Tubulin serves as a loading control.

(**C**) *SMYD2* and *MAPKAPK3* are mostly cytoplasmic in pancreatic cell lines. Immunoblot analysis with the indicated antibodies from cytoplasmic, soluble nucleus, and chromatin fractions of SW1990 and MIA PaCa2 human pancreatic cancer cell lines and primary PDAC cell lines established form *Kras;p53* and *Kras;p53;Smyd2* mutant mice. Tubulin, LSD1 and histone H3 serve as cytoplasmic, soluble nucleus/chromatin, and chromatin fractions controls respectively.

**Supplemental Figure S4. *Smyd2* deletion suppresses lung adenocarcinoma growth and improves survival in a mouse model.**

**(A)** Correlation between higher *SMYD2* expression and decreased survival in non-small cell lung (NSCLC) cancer patients. Kaplan-Meier survival plot with hazard ratio and log rank *P*-value are calculated and plotted using the analysis tool that can be accessed online at kmplot.com/lung (Gyorffy et al. 2013).

**(B)** Representative immunohistochemical analysis of SMYD2 expression (12 independent samples examined) from mouse and human lung adenocarcinoma (LAC) and wild type control lungs (WT). Scale bars, 50 μm.

**(C)** Representative macroscopic picture of lungs, HE staining and IHC for phosphorylated Histone 3 (pH3, a marker of proliferation) from *Kras;p53* and *Kras;p53;Smyd2*.

**(D-F)** Quantification of tumor burden, tumor number and proliferation (pH3). Tumors were analyzed and quantified at 12 weeks post-infection (n = 6 for each experimental group).

**(G)** Kaplan-Meier survival curves of *Kras;p53* (n = 15) and *Kras;p53;Smyd2* (n = 16) mutant mice developing Ras-driven lung tumors. *p* = 0.0039 log-rank test for significance.

n.s.: not significant; *: *p* < 0.05; **: *p* < 0.01; ***: *p* < 0.001; (p value calculated by two-tailed unpaired Student’s t-test; data are represented as mean +/– SEM).

**Supplemental Figure S5. Methylation of MAPKAPK3 by SMYD2 *in vitro*.**

(**A**) MAPKAPK3 K355 is monomethylated *in vitro*. HPLC elution profiles showing expected elutions of K355-containing peptides either unmethylated (upper panel), monomethylated (middle panel) or dimethylated (lower panel) after *in vitro* SMYD2 methylation of MAPKAPK3. Note that deuterated S-Adenosyl-l-methionine was used as a methyl donor and that samples were chemically propionylated prior to trypsin digestion (related to Fig. 2D).

(**B**) MAPKAPK3 K355 is not conserved in MAPKAPK2 and MAPKAPK5. Alignment of human MAPKAPK3, MAPKAPK2, and MAPKAPK5 C-terminal amino acid sequences.

(**C**) MAPKAPK3 K355 is monomethylated *in vivo.* MS/MS spectrum corresponding to MAPKAPK3 K355me1 peptide from HEK 293T cells overexpressing SMYD2 and MAPKAPK3 (related to Fig. 3A). Samples were chemically propionylated prior to trypsin digestion.

(**D**) SMYD2 is required for methylation of MAPKAP3 in human lung cancer cells. Immunoblot analysis with the indicated antibodies after MAPKAPK3 immunoprecipitation using extracts from H358 and H441 cells stably expressing Control or SMYD2 shRNAs. Inputs are shown as loading and shRNA efficiency controls (n = 2).

**Supplemental Figure S6. MAPKAPK3 inhibition decreases cell proliferation and inflammation.**

(**A**) *Smyd2* deletion impairs inflammatory response. Concentration of inflammatory cytokines in the serum of *Kras* and *Kras;Smyd2* mutant mice (n = 3 for each experimental group), as well as wild-type mice (WT , n = 2) animals (6 months of age).

(**B**) Immunoblot analysis of MAPKAPK3 expression from total protein extracts of SW1990 cells after stable transfection with control or MAPKAPK3 shRNA plasmids (related to Fig. 3D). Actin serves as a loading control.

(**C**) MAPKAPK3 inhibition decreases PDAC development. Representative images for HE staining and IHC for pERK1/2, αSMA and CD45 (a marker of leukocytes) on tumor sections from *Kras* mutant mice 7 days after pancreatitis induction with vehicle control or treatment with the PF-3644022 inhibitor (related to Figure 3H). Scale bars, 100 μm.

(**D-E**) Quantification of αSMA and CD45 positive areas in pancreata of *Kras* mutant mice treated with vehicle control or the PF-3644022 inhibitor (n = 5 for each experimental group).

(**F**) MAPKAPK3 inhibition decreases inflammatory response. Immunoblot analysis with the indicated antibodies of pancreatic tissue lysates from *Kras* mutant mice treated with vehicle control or the PF-3644022 inhibitor (n = 5, representative samples are shown).

(**G**) BAY-598 enhances the toxicity of doxorubicin in human SW1990 PDAC cells (related to Fig. 4C). Changes in cell viability (MTT assay, *p* = 0.0014) in response to doxorubicin at various concentrations with (IC50 ~0.25 μM) or without (IC50 ~3.4 μM) BAY-598 co-treatment at 10 μM for 48h. Combined results of three independent experiments performed in triplicates are shown.

*: *p* < 0.05; **: *p* < 0.01; ***: *p* < 0.001 (p value calculated by two-tailed unpaired Student’s t-test; data are represented as mean +/– SEM).

**Supplemental Table S1. List of candidate SMYD2 substrates.**

List of potential SMYD2 substrate candidates detected on two independent protoarray radiolabeled methylation assays.

**Supplemental Reference.**

Gyorffy B, Surowiak P, Budczies J, Lanczky A. 2013. Online survival analysis software to assess the prognostic value of biomarkers using transcriptomic data in non-small-cell lung cancer. *PLoS One* **8**: e82241.
